# Molecular karyotyping and gene expression analysis in childhood cancer patients

**DOI:** 10.1007/s00109-020-01937-4

**Published:** 2020-06-23

**Authors:** Galetzka Danuta, Müller Tobias, Dittrich Marcus, Endres Miriam, Kartal Nergiz, Sinizyn Olesja, Rapp Steffen, Zeller Tanja, Müller Christian, Hankeln Thomas, Scholz-Kreisel Peter, Chorzempa Heather, Mirsch Johanna, Poplawski Alicia, Rossmann Heidi, Spix Claudia, Haaf Thomas, Prawitt Dirk, Marron Manuela, Schmidberger Heinz

**Affiliations:** 1 Department of Radiation Oncology and Radiation Therapy, University Medical Centre, Johannes Gutenberg University Mainz, Obere Zahlbacher Str. 63, 55131 Mainz, Germany; 2 Bioinformatics Department, Julius Maximilians University, Würzburg, Germany; 3 Institute of Organismal and Molecular Evolution, Molecular Genetics and Genome Analysis, Johannes Gutenberg University, Mainz, Germany; 4 Preventive Cardiology and Preventive Medicine, Center for Cardiology, University Medical Centre, Mainz, Germany; 5 University Heart & Vascular Center, Clinic for Cardiology, University Medical Centre Hamburg-Eppendorf, Hamburg, Germany; 6 Dzhk (Deutsches Zentrum für Herzkreislauf-Forschung), Standort Hamburg, Lübeck, Kiel, Hamburg, Germany; 7 Institute of Medical Biostatistics, Epidemiology and Informatics, University Medical Centre, Mainz, Germany; 8 Center for Pediatrics and Adolescent Medicine, University Medical Centre, Mainz, Germany; 9 Institute of Human Genetics, Julius Maximilians University, Würzburg, Germany; 10 Radiation Biology and DNA Repair, University of Technology, Darmstadt, Germany; 11 Institute of Clinical Chemistry and Laboratory Medicine, University Medical Centre, Mainz, Germany; 12 German Childhood Cancer Registry, Institute of Medical Biostatistics, Epidemiology and Informatics, University Medical Centre, Mainz, Germany; 13 Leibniz Institute for Prevention Research and Epidemiology – BIPS, Bremen, Germany

**Keywords:** Childhood cancer, Copy number variation, Radiation, Gene expression, Primary secondary cancer

## Abstract

**Abstract:**

The genetic etiology of sporadic childhood cancer cases remains unclear. We recruited a cohort of 20 patients who survived a childhood malignancy and then developed a second primary cancer (2N), and 20 carefully matched patients who survived a childhood cancer without developing a second malignancy (1N). Twenty matched cancer-free (0N) and additional 1000 (0N) GHS participants served as controls. Aiming to identify new candidate loci for cancer predisposition, we compared the genome-wide DNA copy number variations (CNV) with the RNA-expression data obtained after in vitro irradiation of primary fibroblasts. In 2N patients, we detected a total of 142 genes affected by CNV. A total of 53 genes of these were not altered in controls. Six genes (*POLR3F*, *SEC23B*, *ZNF133*, *C16orf45*, *RRN3*, and *NTAN1*) that we found to be overexpressed after irradiation were also duplicated in the genome of the 2N patients. For the 1N collective, 185 genes were affected by CNV and 38 of these genes were not altered in controls. Five genes (*ZCWPW2*, *SYNCRIP*, *DHX30*, *DHRS4L2*, and *THSD1*) were located in duplicated genomic regions and exhibited altered RNA expression after irradiation. One gene (*ABCC6*) was partially duplicated in one 1N and one 2N patient. Analysis of methylation levels of *THSD1* and *GSTT2* genes which were detected in duplicated regions and are frequently aberrantly methylated in cancer showed no changes in patient’s fibroblasts. In summary, we describe rare and radiation-sensitive genes affected by CNV in childhood sporadic cancer cases, which may have an impact on cancer development.

**Key messages:**

• Rare CNV’s may have an impact on cancer development in sporadic, non-familial, non-syndromic childhood cancer cases.

• In our cohort, each patient displayed a unique pattern of cancer-related gene CNVs, and only few cases shared similar CNV.

• Genes that are transcriptionally regulated after radiation can be located in CNVs in cancer patients and controls.

• *THSD1* and *GSTT2* methylation is not altered by CNV.

**Electronic supplementary material:**

The online version of this article (10.1007/s00109-020-01937-4) contains supplementary material, which is available to authorized users.

## Introduction

Determining genetic risk factors for cancer is a major goal of medical research. The increase of knowledge about genetic risk factors aims to improve cancer diagnostics and, due to therapeutic advances, contribute to increased overall survival for pediatric cancer. As pediatric cancer survivors reach adulthood, the development of secondary malignancies becomes a significant issue for these patients. Treatment of the primary neoplasm with chemotherapy (systemic therapy) and/or radiotherapy has been described as a risk factor for second neoplasms after childhood cancer [1]. As only a small percentage of the treated children suffer from a second neoplasm, other factors are likely to be involved [2]. A predisposition for the occurrence of a second neoplasm in childhood might be a pre-existing somatic genetic variation responsible for, or associated with, DNA-repair, cell cycle control, and other genes crucial for tumor development [3]. Genetic variation among other modifications may manifest as SNP (single nucleotide polymorphism, mutation) or/and chromosomal copy number variation (CNV). Additive, epigenetic modifications, like aberrant methylation may also lead to tumor development.

CNVs may harbor genes and/or regulatory regions that could contribute to complex diseases such as cancer, whose development is triggered and orchestrated by the interaction of many genes. Two consecutive chromosomal aberrations can be described in neoplasias: Primary somatic variations as initiating events and secondary aberrations that are acquired during transformation toward cancer [4]. Typically, chromosomal abnormalities that accumulate during tumor evolution lead to an unbalanced genome. On the other hand, balanced chromosomal alterations are often associated with cytogenetically cryptic deletions or duplications in the breakpoint regions [5]. These alterations can have a direct effect on transcript levels and thus gene expression [6]. So far there have been few studies on primary fibroblasts of cancer patients to study underlying predisposing genomic variations and associated gene expression changes. Fibroblasts of breast and thyroid cancer patients were almost always found to have defective DNA repair and/or cell cycle regulation [7]. Abnormal gene expression in the somatic cells of unaffected parents of retinoblastoma patients is also consistent with an inherited predisposition to cancer development [8].

Radiation and chemotherapeutic agents do not mechanistically distinguish a tumor cell from healthy tissue and the application of these genotoxic agents may be another source of acquired CNVs. Frequent induction of chromosomal aberrations after irradiation has been reported by Massenkeil et al. [9] in skin fibroblasts in vivo*.*

To protect the healthy tissue from damage, it is especially important to understand the molecular mechanisms involved in the cellular response to radiation. Several attempts have been undertaken to analyze the transcriptional effects after irradiation of different tissues or cells, with different cell culture conditions, doses, and time points. Furthermore, it remains unclear whether DNA duplications or deletions are associated with the formation of epigenetic alterations, such as DNA methylation, which could play a role in cancer development [10].

To identify genomic susceptibility factors for primary and secondary cancer formation in childhood, we compared molecular cytogenetic profiles by SNP array analysis in primary fibroblasts of childhood cancer survivors (1N) and carefully matched patients with second cancer (2N), alongside with cancer-free controls (0N). We determined the gene expression profile in primary fibroblasts in vitro after X-ray treatment and correlated it with the genes located within the deletions and duplications detected by SNP array analyses. Specifically, we tested the hypothesis that the occurrence of secondary cancer is associated with modifications in the expression of cell cycle control and DNA repair pathways. Finally, we analyzed the methylation patterns in the putative promoter regions of two candidate cancer-relevant genes, which resided within CNV regions and displayed differential expression after irradiation.

## Material and methods

### Patient collective

This study was approved by the Ethics Committee of the Medical Association of Rhineland-Palatinate (no. 837.440.03 (4102) and no. 837.262.12(8363-F)). With the help of the German Childhood Cancer Registry, 20 individuals who survived a childhood malignancy and then developed a second primary cancer (2N) and 20 carefully matched (first tumor, manifestation age, sex) individuals who survived a childhood cancer without developing a second malignancy (1N) were recruited for the KiKme study (Cancer in Childhood and Molecular Epidemiology). Twenty matched patients (sex and age) without cancer from the Department of Accident Surgery and Orthopedics in Mainz Germany served as controls (0N). Written informed consent to use fibroblasts for research purposes was obtained after genetic counseling for all participating patients. No patient had intellectual disability or any other severe mental disease based on the clinical impression and personal history.

The numbering of the patients does not represent the recruiting number and was chosen randomly. Skin biopsies were taken at the earliest 2 years after the last cancer therapy. Eleven patients suffered from acute lymphatic or myeloid leukemia, 5 patients from Hodgkin or Burkitt lymphoma, and 4 patients from other solid tumors as primary malignancy. The second cancers in the 2N group included myelodysplastic syndrome, lymphoma, thyroid cancer, and other solid tumors. All patients were followed up from primary cancer diagnosis to the time when they were recruited. With the exception of one patient (1N), all patients received chemotherapy, radiotherapy, or combination therapy. Six patients received allogeneic bone marrow transplantation. Clinical data of the participating patients are shown in Table 1.

**Table 1:** Clinical details of the 2N and 1N cancer patient groups

Tumors		2N		1N	
		First	Second	First	Second
Age at diagnosis of cancer (year)		7.2 ± 4.3	15.2 ± 5.9	7.5 ± 4.4	
Tumor entity	Acute lymphatic/myeloid leukemia	11	1	9	-
	Hodgkin or Burkitt lymphoma	5	5	5	-
	Solid tumors	4	14	6	-
Age at fibroblast collection (year)		27.1 ± 3.7		26.3 ± 4.4	
Treatment					
CT−RT−		1		4	
CT+RT−		3		2	
CT−RT+		3		4	
CT+RT+		13		10	

*CT*, chemotherapy; *RT*, radiation therapy including radioiodine therapy for papillary thyroid cancer

Although it was reported that 7–8% of children affected by cancer carry an unambiguous predisposing germline variant, predominantly within *TP53* and *BRCA2* [11], no proven pathogenic germline variants in *TP53*, *BRCA1*, or *BRCA2* were identified using Sanger sequencing applying the ACMG criteria, in our cohort (Mutation Databases: (http://p53.iarc.fr/; https://www.ncbi.nlm.nih.gov/clinvar/ and https://www.lovd.nl/)). In one case, an *RB1* oncogenic splice mutation was detected. This patient was excluded from further analysis. The remaining patients did not fit the criteria of an inherited childhood cancer syndrome [12].

Currently, there are several attempts to characterize the common CNVs which have no impact on disease. The 1000 Genomes project (http://www.1000genomes.org), the genome of the Netherlands project (http://www.nlgenome.nl), and the Toronto Database of Genomic Variants (http://dgv.tcag.ca/) are examples of these attempts. Since the boundaries of the variants are not well defined, there could be an over-estimation in the actual size of the variants. In addition, some ethnic aspects may contribute to the prevalence of a specific CNV. Therefore, we compared the cancer patients’ CNV with the data of matched 0N controls and 1000 0N cases without cancer, diabetes, obesity, dyslipidemia, and stroke from the Gutenberg Heart Study (GHS), which had the advantage that the samples were analyzed in the same laboratory with the same technique and the participants’ samples came from similar ethnic backgrounds. The aim was to detect genes which were affected in the cancer patients but not in the controls, which may therefore be considered as rare, putative, and predisposing variations. The GHS is a community-based prospective, observational single-center cohort study in the Rhein-Main-Region in western Mid-Germany. The GHS has been approved by the local ethics committee and by the local and federal data safety commissioners. The primary aim of the GHS study is to evaluate and improve cardiovascular risk stratification.

### Cell culture and experimental procedure

Primary fibroblasts from skin biopsies were cultured in DMEM (Invitrogen, Karlsruhe, Germany) and were supplemented with 15% fetal bovine serum (FBS) (Biochrom, Berlin, Germany), 1% vitamins, and 1% antibiotics (Pen/Strep) (Biochrom, Berlin, Germany) at 37 °C and 5% CO_2_. All experiments using the primary fibroblasts were performed with growth-arrested cells in the G0/G1 stage in 10-cm cell culture dishes. Confluency of the cells was achieved by contact inhibition and subsequent cultivation for 2 weeks. Over 90% of the cells were in the G0/G1 stage of the cell cycle which was confirmed by FACS (flow cytometric cell cycle analysis). For comparisons of 0N, 1N, and 2N patients, fibroblasts with similar passages 9 (± 2) were used. Cells were exposed to X-rays with a D3150 X-Ray Therapy System (Gulmay Ltd., Surrey, UK) at 140 kV and a dose rate of 3.62 Gray (Gy)/min at room temperature. Sham-irradiated cells were kept at the same conditions in the radiation device control room. Cells were exposed to single doses ranging from 2 to 8 Gy and were returned to the incubator. Cells were harvested by a brief treatment with trypsin/EDTA (Biochrom, Berlin, Germany) and washed with PBS (–Mg/–Cl) at 15 min, 2 h, and 24 h after irradiation. Resulting pellets were stored at − 80 °C until DNA or RNA preparation. Cell lines MCF7 (ATCC, Manassas, VA, USA), ZR-75-1, EFO27, and T47D (ATCC, Manassas, VA, USA) were cultivated in RPMI1640 (Gibco) supplemented with 10% FBS, 2.5% HEPES buffer (Sigma), and 1% antibiotics (Pen/Strep) (Life Technologies). The A549 (ATCC, Manassas, VA, USA) cell line was cultured in DMEM modified with 10% FBS.

### SNP array (molecular karyotype analysis)

Molecular karyotyping (SNP array) was performed using DNA (isolated with the NucleoSpin tissue kit Macherey-Nagel, Germany) from untreated primary fibroblasts in passage 5 (2N, 1N, 0N). High-resolution screening for microdeletions and duplications was performed with the Affymetrix GeneChip Genome-Wide Human SNP array 6.0 and the GeneChip Genome-Wide SNP Sty Assay Kit 5.0/6.0, following the protocol developed by the manufacturer (Affymetrix, Santa Clara, CA, USA). Data calculation was performed with Affymetrix Genotyping Console 4.2.0.26 and Chromosome Analysis Suite 3.1.0.15. The segment filters for gains and losses were set at a minimum of 5 markers and 20 kb. All samples passed the QC control filters (MAPD_<0.25, SNPQC>_15.00, Waviness SD <_0.12).

### RNA-sequencing, data analysis, and statistics

Total RNAs were prepared from treated and untreated fibroblast cultures using the Nucleo Spin RNA Plus Kit from Macherey-Nagel. The RNA integrity was assessed with a Bioanalyzer 2100 (Agilent RNA 6000 Nano Kit, Agilent Technologies, Santa Clara, USA). One microgram of total RNA (QuBit, Thermo Fisher Scientific) RIN ≥ 8 was used for library construction using the TruSeq RNA Sample Prep Kit v2 (Set A and B, Illumina) following the manufacturer’s instruction. RNA-Seq libraries were pooled, cBot clustered, and sequenced on a HiSeq2500 instrument (Illumina) in high-output mode. Reads with a length of 50 nucleotides were generated using TruSeq SR (single read) Cluster Kit v3 (Illumina) and TruSeq SBS Kit v3 (Illumina). Data was generated by RTA Version 1.8.4 (real-time analysis) and converted to FASTQ format using bcl2fastq Version 1.8.4. (Illumina).

Raw reads were cleaned from adapter sequences using Trimmomatic. Cleaned reads were aligned to the human reference genome (GRCh38) using STAR. Expression per gene expressed as the number of aligned reads per gene was quantified using FeatureCounts. Data analysis was performed using R with 51 samples varying in the dose of applied radiation (0 Gy, 2 Gy, 5 Gy, 8 Gy) and time post-irradiation (15 min, 2 h, 24 h) for analysis. Genes with less than 10 counts in 4 samples were discarded. Data were normalized for sequencing depth using the EdgeR package. Transformation to log2 counts per million was performed via the Voom method, implemented in the limma-package. Differential gene expression dependent on dose and time points was detected using linear models implemented in the limma-package. Genes with an adjusted *p* value smaller than 0.05 were flagged as significant for further analyses. *p* values were adjusted for false discovery rate (FDR) (Benjamini-Hochberg procedure).

### Quantitative real-time PCR for gene expression and copy number variation

Total RNAs were prepared from treated and untreated fibroblast cultures using the Nucleo Spin RNA Plus Kit from Macherey-Nagel. Two micrograms of the RNA samples was reversely transcribed into cDNA using the SuperScript IV First-Strand random hexamer Synthesis System (Invitrogen). Genomic DNA was isolated with the NucleoSpin tissue kit (Macherey-Nagel, Germany). Forward and reverse primers (Exon-spanning for gene expression) were designed with the Primer-Blast program (https://www.ncbi.nlm.nih.gov/tools/primer-blast/). *RRN18S* and *TBP* for gene expression and *HEM3* and *RFC3* for copy number calculations were used as endogenous control genes (Online Resource Table: Primer sequences). Each 10-μl reaction volume contained 25 ng cDNA or DNA template in 5 μl Sybr-Green Master Mix (Roche), 2 μl RNase-free PCR graded water (Roche), and 1 μl each of forward and reverse primer (10 μM). All reactions were performed in triplicate and in two stages, with one cycle of 95 °C for 10 min (first stage) and 45 cycles of 94 °C for 10 s, (TM-primer)°C for 10 s, and 72 °C for 10 s (second stage) using the LightCycler 480II Roche. Amplification qualities were assayed using melting curves and agarose gel analysis. The qPCR amplification efficiency was calculated using the LingReg program and the CT values were corrected using the mean amplification efficiency. Relative quantification was carried out with the ΔΔCT method using the two endogenous control genes and the control 0 Gy or 0N probands for calibration. Statistical analyses were conducted using the unpaired *t* test. Expression changes with *p* value < 0.05 were considered significant.

### Bisulfite pyrosequencing

Genomic DNA was isolated with the NucleoSpin tissue kit (Macherey-Nagel, Germany). Bisulfite conversion of 0.2 μg DNA was performed with the EpiTect Bisulfite Kit (Qiagen, Hilden, Germany) according to the manufacturer’s instructions. PCR and sequencing primers for the analyzed genes were designed with PyroMark Assay Design 2.0 software (Qiagen) (online Resource Table: Primer sequences). The 25 μl PCR reactions consisted of 2.5 μl 10x PCR buffer, 20 mM MgCl_2_, 0.5 μl dNTP mix (10 mM), 1 μl of each forward and reverse primer (10 μM), 0.2 μl FastStart Taq DNA Polymerase (5 U/μl) (Roche Diagnostics, Mannheim, Germany), 18.8 μl PCR-grade water, and 1 μl (~ 100 ng) bisulfite-converted template DNA. PCR amplifications were performed with an initial denaturation step at 95 °C for 5 min, 35 cycles of 95 °C for 30 s, 55 °C for 30 s and 72 °C for 45 s and a final extension step at 72 °C for 5 min. Bisulfite pyrosequencing was performed on a PyroMark Q96 MD Pyrosequencing System using the PyroMark Gold Q96 CDT Reagent Kit (Qiagen) and 0.5 μl of sequencing primers (10 mM). Data analysis was performed with the Pyro Q-CpG software (Qiagen).

### FISH analysis

Metaphase chromosome spreads of the patients were prepared from primary mitotic fibroblasts. BAC clones (RP11-139D07 for the 2N4 and RP11-327M19 for the 2N7 patient) were selected from the Wellcome Trust Sanger Institute Ensembl contigs and obtained from the Resource Center Primary Database of the German Human Genome Project and ResGen (Invitrogen). Genomic BAC DNAs were labeled with Tetramethyl-rhodamine-5-dUTP* (Roche) or 25 nmol Fluorescein-12-dUTP* (Roche) by standard nick-translation and FISH-mapped on metaphase chromosomes. Control BAC clones were chosen for 16q terminal or 2q terminal chromosome areas. Images were generated using the Leica microscope CTR MIC and Software CW4000.

## Results

### Molecular karyotype analysis (SNP array) of 2N, 1N, and 0N controls

The concept of the study was to detect genes that were affected in the cancer patients (2N, 1N) but not in the controls (0N and 1000 GHS), which may, therefore, be considered as a rare, putative, and predisposing variation. We detected rare germline CNVs in eighteen 2N and sixteen 1N patients. In some cases, the detected aberrations in the SNP array analysis overlapped between patients and controls. For the final compendium of putative pathogenic aberrations, we selected only genes that were not affected in the control section, but the annotation of the aberration reflects the complete duplicated or deleted CNV area. Altogether we detected 142 affected genes in 2N patients of which 53 genes were not altered in controls (matched 0N and 1000 GHS). For the 1N collective, there were 185 genes affected by CNVs of which 38 genes were uniquely altered in the 1N cancer patients. Interestingly, 22 genes in 2N patients within CNV and eighteen genes in 1N have previously been described to be associated with tumor development, growth, apoptosis, and chromosomal stability, or as differentially expressed in cancer. (TCGA data base: https://www.cancer.gov/about-nci/organization/ccg/research/structural-genomics/tcga). Only one gene (*ABCC6*) was partially duplicated in two matched patients (1N5 and 2N4) and duplicated in five out of 1000 controls (2N; arr[hg19] 16p13.11(15,048,755-16,295,900)x3 and 1N; arr[hg19] 16p13.11(16,294,705-16,798,651)x3). Both patients suffered from leukemia and patient 2N4 later developed a slow-growing brain tumor. Each 2N patient displayed a unique CNV pattern which was not seen in other patients of the 2N group. Altogether we detected sixteen heterozygous and one homozygous duplication, as well as eleven heterozygous and one homozygous deletion in the 2N group. The homozygous deletion affected the *TPTE2P3* gene, which is classified as a pseudogene with expression restricted to the testis [13]. The findings for the 1N patient group were similar to the observed ones in 2N patients. Here we also detected unique CNV patterns, with the exception of three regions: 19q13.42(54,716,827-54,741,307)x3, 22q11.21(21,567,218-21,845,282)x3, and 14q11.2(24,431,136-24,499,742)x1 which were duplicated in more than one 1N case. The duplication in chromosome 19q13.42 contains the *LILRB3* gene and occurred in two leukemia cancer patients, whereas the duplication in 22q11.21 encompasses five genes (*HIC2*, *PI4KAP2* (pseudogene), *POM121L8P* (pseudogene), *RIMBP3B*, and *RIMBP3C*). The patients carrying this aberration suffered from leukemia and solid tumors. The aberration 14q11.2(24,431,136-24,499,742)x1 contains the *DHRS4L2* gene, which is downregulated after radiation and occurred in our patients with leukemia and solid tumor. In total, we detected thirteen heterozygous, one homozygous duplicated, and twelve deleted heterozygous regions in the 1N patient cohort.

The CNVs did not always affect a whole gene. We detected intronic deletions in *IGSF21*, *NCK1*, and *MCU*, and intronic duplications in the *RBFOX3*, *COL11A*, *SORCS1*, *FMNL2*, and *NLGN1* genes. These sites harbor transcription factor binding regions. In total, seven pseudogenes and eight microRNAs were affected. Six long coding (LINCO) and two anti-sense RNAs (AS) were affected by CNVs in cancer patients, but not in controls (see Tables 2 and 3 and for more detailed information Online Resource 1, Table: 1N CNV; 2N CNV)*.*

**Table 2:** Rare germline CNV’s detected in 16 of 20 patients suffering from one cancer (1N). Genes indicated in bold are cancer related (Pubmed 2019; see further information in Online Resource 1)

Patients 1N	Type	Chromosome position [hg19]	Number of genes/probes	Genes	1000 GHS controls
1	Del	4q22.3(98,338,184-98,360,773)x1	1/16	*STPG2-AS1*	0
2	Del	4q31.21(141,590,313-141,612,737)x1	1/25	*TBC1D9*	0
3	Dupl	3p24.1(28,355,385-28,522,017)x3	3/65	*AZI2*, *CMC1*, *ZCWPW2*	0
4	Dupl	16p13.11(16,294,705-16,798,651)x3	5/30	*PKD1P1 (pseudogene), MIR3179-2*, *MIR3670-2*, *MIR3180-2*, *MIR6511A2*	0
5	Del	8q11.21q11.23(50,048,130-53,211,910)x1	3/2006	***PCMTD1***, *PXDNL*, ***ST18***	0
6	Dupl	9p24.1(5,221,817-5,408,358)x3	2/145	***PLGRKT***, ***INSL4***	0
7	Del	17p13.3(513-38,924)x1	1/19	***DOC2B***	0
	Del	4q22.1(88,176,912-88,295,008)x1	2/61	*HSD17B11*, ***HSD17B13***	0
8	Dupl	10q25.1(108,746,879-108,777,331)x4	1/25	*SORCS1 (intron)*	0
	Del	11q14.3(89,438,613-89,461,619)x1	1/19	*TRIM77*	2
	Del	16q23.1(78,057,102-78,117,280)x1	1/48	*CLEC3A*	2
9	Dupl	6q14.3(86,136,432-86,346,699)x3	3/87	***NT5E*** *,* ***SNX14***, ***SYNCRIP***	0
	Del	10q22.1(74,515,548-74,560,343)x1	1/23	***MCU*** *(intron)*	0
	Del	19p12(22,159,002-22,179,901)x1	1/10	*ZNF208*	0
	Del	6p24.3(10,468,665-10,535,651)x1	1/78	***GCNT2***	5
10	Dupl	3q26.31(173,247,466-173,289,668)x3	1/23	*NLGN1 (intron)*	2
11	Dupl	3p21.31(47,799,742-47,849,936)x3	2/50	***DHX30***, ***SMARCC1***	0
	Dupl	21q22.12(37,481,942-37,617,188)x3	3/96	***CBR3-AS1***, ***CBR3***, *DOPEY2*	0
12	Del	2p21(45,312,361-45,504,006)x1	1/170	***LINC01121***	0
13	Dupl	13q14.3(52,923,759-53,053,990)x3	3/47	***CKAP2***, ***THSD1***, ***VPS36***	0
	Dupl	2q23.3(153,212,500-153,273,886)x3	1/54	***FMNL2 (intron)***	0
14	Dupl	6q16.2q16.3(100,277,839-100,643,681)x3	1/204	*MCHR2*	0
15	Del	19q13.12(35,774,555-35,795,979)x1	2/14	*HAMP*, *MAG*	0
5, 3	Dupl	19q13.42(54,716,827-54,741,307)x3	1/26	*LILRB3*	2
16, 14	Dupl	22q11.21(21,567,218-21,845,282)x3	5/35	*HIC2*, *PI4KAP2 (pseudogene)*, *POM121L8P (pseudogene)*, *RIMBP3B*, *RIMBP3C*	4
4, 5	Del	14q11.2(24,431,136-24,499,742)x1	1/49	*DHRS4L2*	0

**Table 3:** Rare germline CNV’s detected in 16 of 20 patients with two independent cancers (2N). Genes indicated in bold are cancer related (Pubmed 2019; see further information in Online Resource 1)

Patients 2N	Type	Chromosome position [hg19]	Number of genes/probes	Genes	1000 GHS controls
1	Del	7q11.21(63,526,244-63,784,883)x1	5/159	*ZNF727*, *ZNF735*, *ZNF679*, *ZNF736*, *ZNF83*	0
	Dupl	Xp11.23(47,859,614-47,960,125)x3	2/69	*ZNF182*, *ZNF630*	6
2	Dupl	1p36.13(17,594,883-17,622,897)x3	1/26	*PADI3*	3
3	Dupl	16q23.1(75,539,395-75,575,997)x4	2/20	*CHST5*, *TMEM231*	7
4	Dupl	16p13.11(15,048,755-16,295,900)x3	10/682	*MPV17L*, ***ABCC1***, ***FOPNL***, ***MYH11***, ***NDE1***, ***NTAN1***, *Corf45* **,** ***RRN3***, ***KIAA0430***, ***MIR484***	0
	Del	1p36.13(18,578,074-18,601,898)x1	1/26	*IGSF21 (intron)*	0
	Del	3p21.31(46,763,773-46,859,820)x1	1/56	*PRSS45*	1
	Del	3q22.3(136,596,428-136,633,070)x1	1/17	*NCK1 (intron)*	0
	Del	7p22.1(6,776,023-6,919,827)x1	1/32	*PMS2CL (pseudogene)*	0
	Dupl	4q31.1(139,830,003-139,974,328)x3	2/77	***NOCT***, *LOC105377448*	0
	Del	2q21.1(132,395,403-132,425,713)x1	1/17	*LINC01087*	0
5	Dupl	17q25.3(77,363,500-77,389,101)x3	1/20	***RBFOX3 (intron)***	6
6	Dupl	9p13.3p13.2(36,229,595-36,728,544)x3	3/235	***MELK***, ***RNF38***, ***GNE***	0
	Dupl	12q13.12(50,619,763-50,670,065)x3	2/24	***LIMA1***, ***MIR1293***	0
7	Dupl	2q11.1(95,415,055-95,777,315)x3	4/108	*ANKRD20A8P (pseudogene)*, ***MAL***, *MRPS5*, ***TEKT4***	0
8	Dupl	2q13(112,118,311-112,475,467)x3	1/129	***MIR4435-2HG***	0
9	Dupl	15q25.1(80,104,017-80,175,045)x3	1/67	*MTHFS*	0
	Dupl	19q13.41(52,974,191-53,127,459)x3	5/102	*ZNF83*, *ZNF137P (pseudogene)*, ***ZNF578***, *ZNF701*, *ZNF808*	0
10	Dupl	19q13.32(46,693,400-46,782,606)x3	2/41	*IGFL1*, *LOC93429*	0
11	Dupl	9q34.3(138,154,269-138,309,335)x3	1/114	*C9orf62*	0
12	Dupl	1q24.2(167,549,279-167,602,533)x3	1/46	***RCSD1***	0
	Del	2q32.1(186,847,769-186,953,324)x1	1/71	***LINC01473***	0
13	Dupl	Xp22.2(13,089,235-13,621,642)x3	4/370	***EGFL6***, ***ATXN3L***, *LINC01203*, *GS1-600G8.3*	3
14	Del	16p13.3(6,797,814-6,835,414)x1	1/65	***RBFOX1***	7
	Del	13q14.3(53,071,139-53,145,646)x0	1/7	*TPTE2P3 (pseudogene)*	1
	Del	9q33.1(118,454,941-118,598,371)x1	1/107	*LOC101928775*	0
	Del	17q22(50,562,850-50,858,462)x1	1/220	*LINC01982*	0
15	Dupl	20p11.23(18,149,194-18,500,918)x3	7/266	*DZANK1*, *CSRP2BP*, *POLR3F*, ***SEC23B***, ***RBBP9***, ***ZNF133***, *MIR3192*	0
	Dupl	11q25(134,347,973-134,719,556)x3	1/346	*LOC283177*	4
	Del	18q22.3(69,388,518-69,587,022)x1	1/158	*LINC01899*	0
16	Del	7q11.23(76,179,701-76,303,498)x1	2/46	*POMZP3*, *LOC100133091*	0

### qPCR and FISH analysis to confirm CNV

**Fig. 1:**
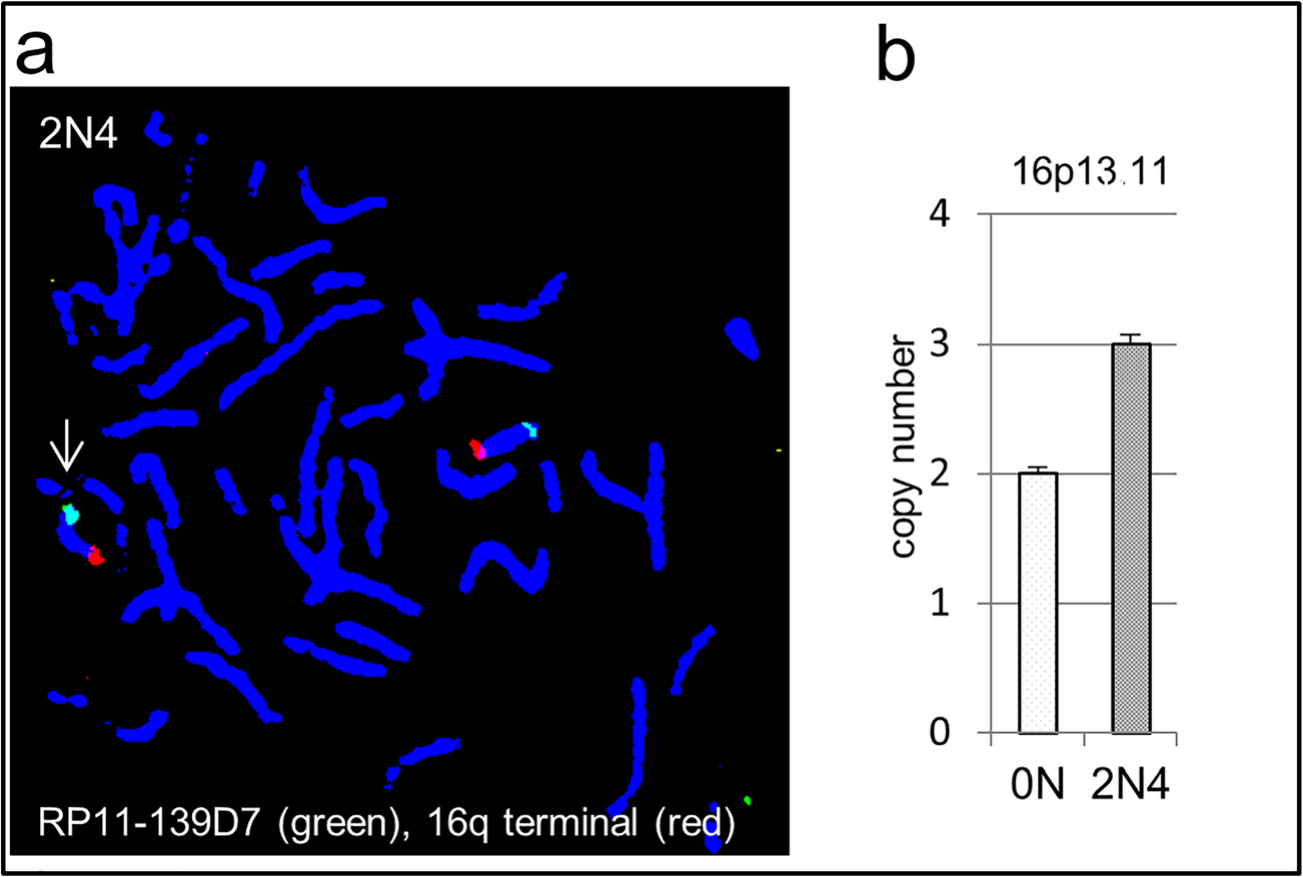
FISH analysis of duplications in 16p13.11 (2N4) and 2q11.1 (2N7). Possible tandem duplication in one 2N cancer patient (with brighter signals than on corresponding chromosomes indicated with white arrow) (**a**). Copy number qPCR analysis may indicate duplications in a mosaic state (**b**)

**Fig. 2:**
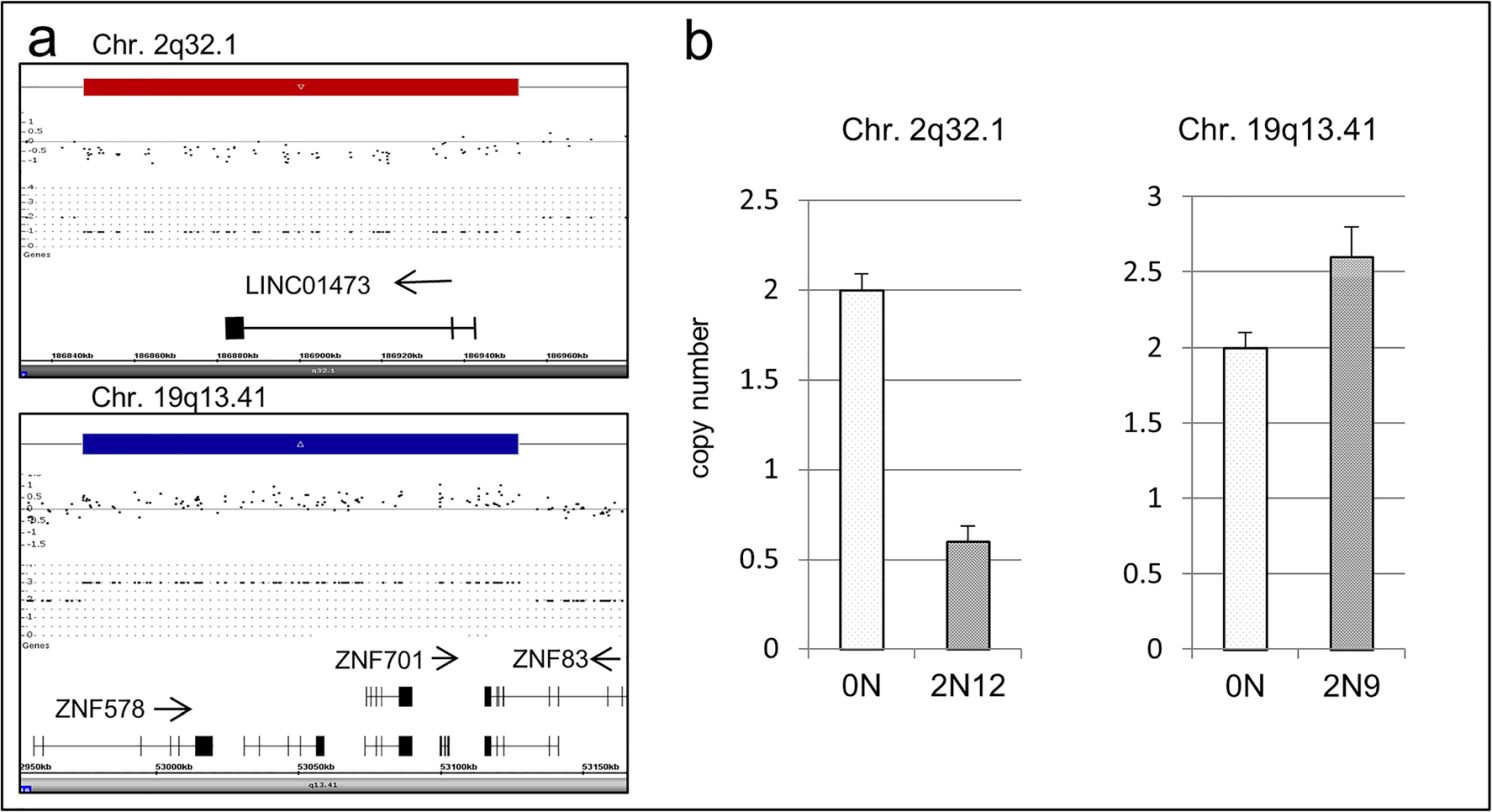
SNP array copy number analysis (**a**) and verification using a qPCR approach (**b**). Case 2N12 shows a deletion in chromosome 2q32.1 and case 2N9 shows a duplication in 19q13.41. qPCR analyses with specific primers show a heterozygous/homozygous deletion

To confirm the results of the SNP array analyses, we chose exemplary regions for verification by qPCR and FISH analysis. In all explored cases, the qPCR result confirmed the duplications detected by the SNP array and the hybridization using specific probes (FISH) indicates tandem duplication in both cases (2N4; 2N7) analyzed. In Fig. 1, the duplication in 2N4 is shown. Since the qPCR technique is suitable for screening for aberrations even in a mosaic state, further verification was conducted using qPCR. Case 2N12 displayed a deletion in chromosome 2q32.1. qPCR analysis with specific primers for this region suggests a heterozygous deletion, whereas the duplication in 19q13.41 in case 2N9 may be a mosaic (Fig. 2).

### Analysis of common CNVs and transcription factor binding sites in controls (0N and 1000 CGH) and patient’s gene free areas

Our analysis of frequently altered regions in the human genome (Online Resource 1, Table: Suppl. common CNV) revealed the presence of transcription factor binding sites either within gene-loci (e.g., the *DUSP22* gene) or within previously described immune response regulating areas (IGK, immunoglobulin kappa locus; IGH immunoglobulin heavy locus, etc.). Our hypothesis is that there may be several areas altered in the patients which do not contain genes, but enhancer-/transcription binding- or CpG-sites that might be important for the regulation of genes outside of CNVs. On these grounds, we conducted an analysis on parts in the genome which are classified to date as gene free (data base UCSC https://genome.ucsc.edu/cgi-bin/hgGateway) and were found to be homozygously or heterozygously deleted mainly in the tumor patients (Online Resource 1 Table: Gene free CNV 2N and 1N). We did not detect any homozygously deleted sites, whereas twelve regions were heterozygously deleted in 2N cases and six in 1N tumor cases. Eight areas were duplicated (six in 2N and two in 1N). Only four of these regions did not contain any transcription factor binding site. Other regions like 5q21.2(103,509,767-103,534,114)x1, harboring *MYC*, *RAD21*, or *SMC3* binding sites, suggest some involvement in the regulation of DNA repair or growth control. There were no significant differences or overlaps between 1N and 2N cases. None of the detected areas contained CpG islands (https://genome.ucsc.edu/).

### Gene expression in cells of 0N patients after irradiation

As stated by other researchers, low background radiation and therapeutic radiation treatments are important inducers of cancer and secondary independent cancers. To estimate the influence of CNVs on gene regulation after irradiation, we designed a study to analyze collateral radiation damage, aiming to detect genes that are transcriptionally altered after irradiation and affected in patients by CNVs.

To compare the gene expression with the genomic gains and losses of former tumor patients, it was necessary to generate a comparable gene expression data set. There are several studies published on gene expression after gamma radiation in human primary fibroblasts, mostly performed using chip technology and examining the expression in 2D and generally at 80% confluency. As G0/G1 is probably the predominant cell cycle stage in collaterally irradiated tissues, we designed our experiments in cell cycle arrested cells, to omit mitotic gene expression and DNA repair in dividing cells, as previously published with skin fibroblasts and neonatal foreskin cell lines [14, 15]. To ensure a wide spectrum of the gene induction after radiation, we used three independent 0N fibroblast cell lines and extracted the RNA after 15 min (early response), 2 h (mid response), and 24 h (late response). The radiation doses were chosen either to be therapeutically relevant (2 Gy) or experimental (5 Gy and 8 Gy). Using the entire data set, without regard for the differences in radiation dose and time, we detected 21,459 dysregulated genes (*p* value < 0.05) post-radiation. After stratifying the results for false discovery rate (FDR) < 0.05, we found 2619 genes to be altered in their transcription rate (Online Resource 2). Considering the post-radiation time, the gene expression varied strongly between 15 min, 2 h, and 24 h. After 15 min, we detected only one regulated gene (*ANAPC4*) (FDR < 0.05) in comparison with gene induction after 2 h (FDR < 0.05; 1472 regulated genes) and 24 h (FDR < 0.05; 1567 regulated genes).

**Fig. 3:**
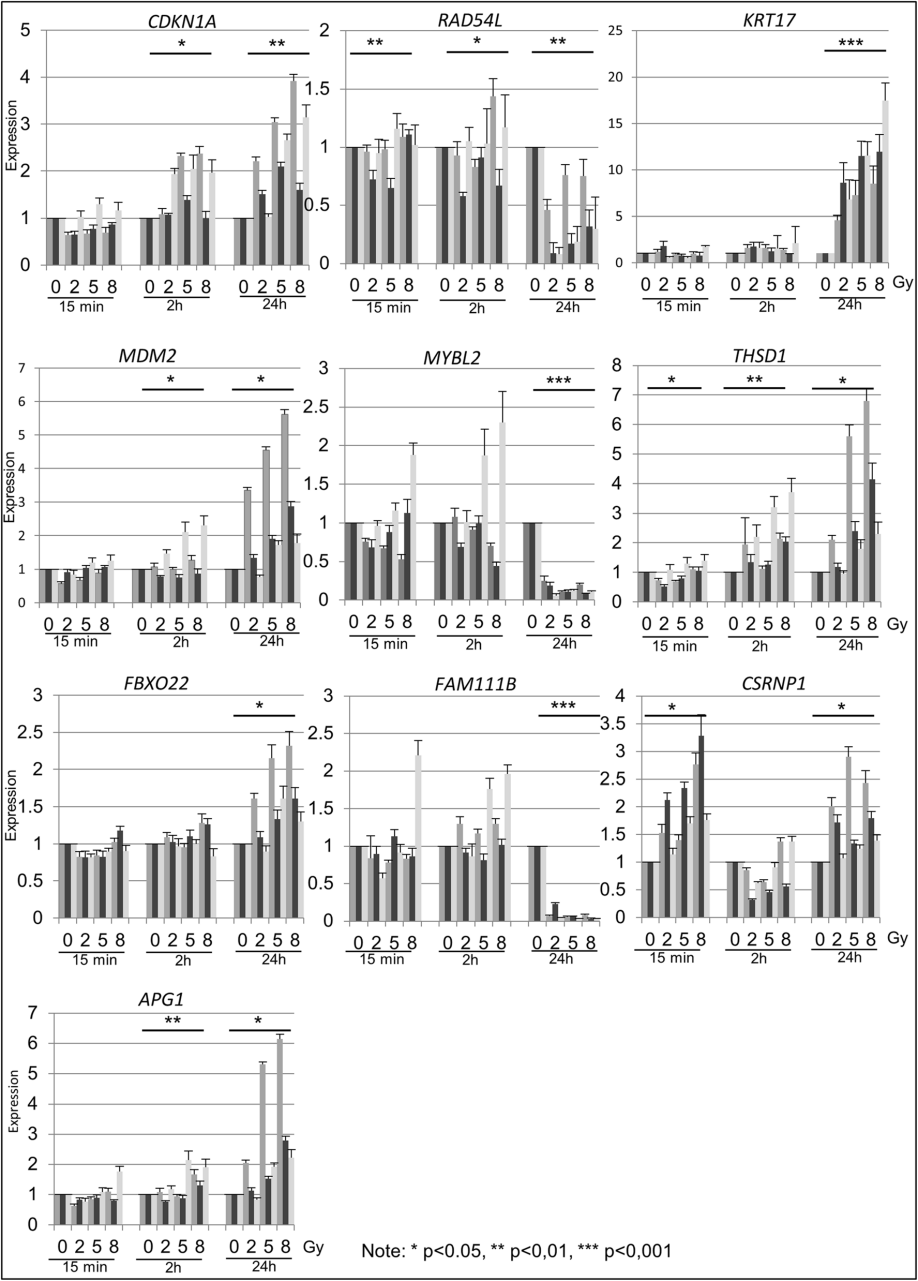
QPCR experiments in three independent 0N cell lines. The analysis of ten genes was performed with irradiated (2, 5, 8 Gy) and control (0 Gy) primary fibroblast cell lines at 15 min, 2 h, and 24 h post-treatment. Significant differences in expression are indicated by asterisk

For the verification of the RNA-seq experiments, we chose ten representative genes (*APG1*, *CDKN1A*, *CSRNP1*, *FAM111B*, *FBXO22*, *KRT17*, *MDM2*, *MYBL2*, *RAD54L*, and *THSD1*) for further characterization using qPCR. Among them are known marker genes that have already been described to be regulated upon radiation, such as *CDKN1A*, *RAD54L*, and *KRT17* (Fig. 3**).**

As stated by Christmann and Kaina [16], mammalian cells express DNA repair genes at a detectable basal level and just a slight upregulation or downregulation may significantly ameliorate the repair capacity of the cell. By convention, an expression change of ± 1.5–2-fold is considered to be biologically relevant. In our experience, the calculation of the fold changes depends on the platform used to generate the data and the bioinformatics normalization approach. Therefore, we used a data set based on the FDR value < 0.05 with 2619 genes (Online Resource 2), with no regard to time and dose (duplicate genes were removed) for comparison with the molecular karyotypes of the patients.

### Irradiation sensitive genes affected by CNVs in cancer patients

**Fig. 4:**
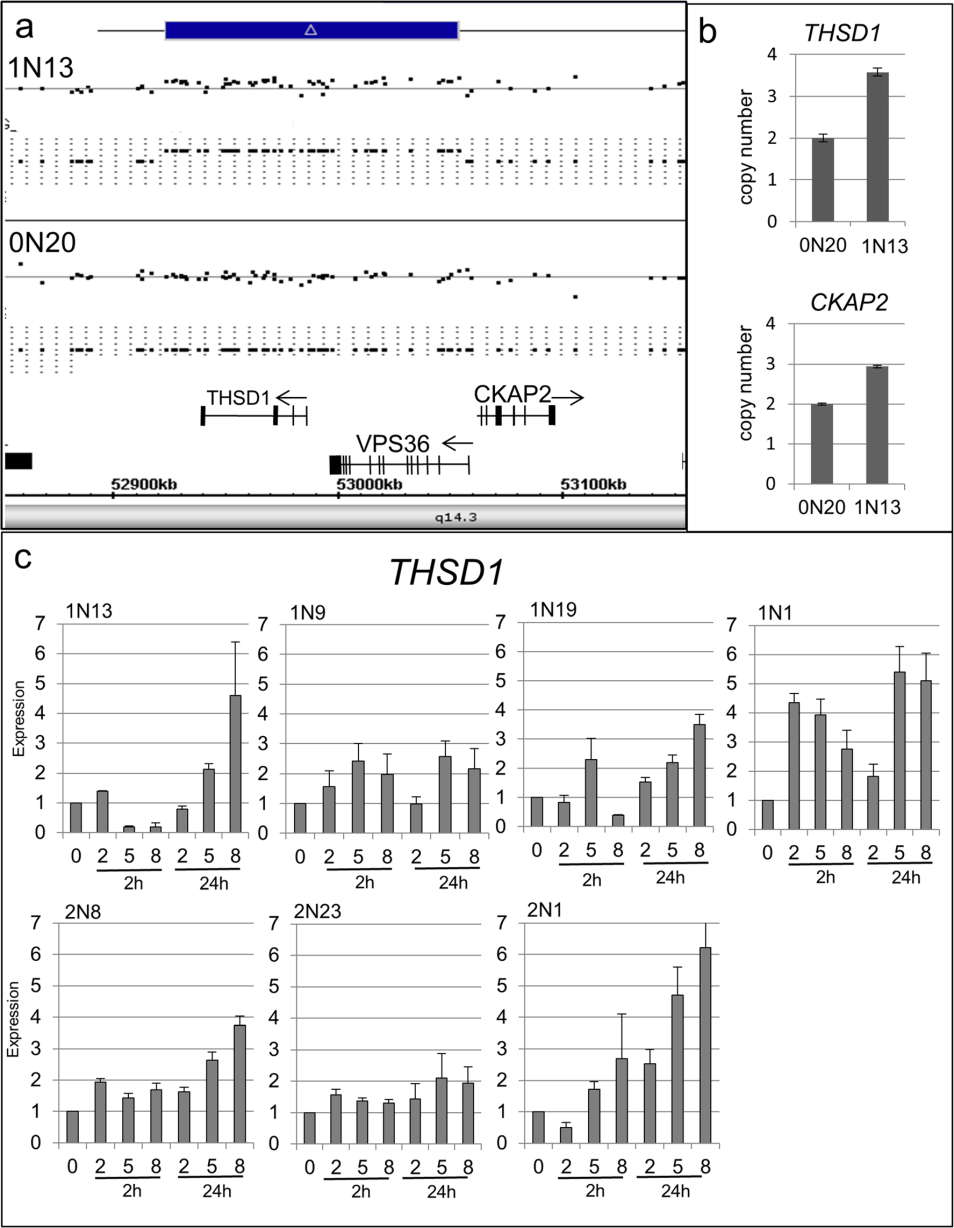
Analysis of the duplication and gene expression of the *THSD1* gene. The scheme shows duplication in 13q14.3 including the *THSD1* gene (**a**). qPCR copy number analysis of this region reveals a heterozygous duplication of the DNA fragment in 1N13 (**b**). RNA expression analysis using qPCR in index patient 1N13 and three independent 2N and 1N cell lines. The analysis was performed with irradiated (2, 5, 8 Gy) and control (0 Gy) cells at 2 h and 24 h post-treatment (**c**)

To analyze the impact of radiation-induced genes in unique patient-related CNVs, we compared the SNP array data with the gene expression signatures obtained after irradiation. Among the 2N patients, we detected six genes (*POLR3F*, *SEC23B*, *ZNF133*, *C16orf45*, *RRN3*, and *NTAN1*) which were overexpressed after irradiation and were duplicated in the genome of ALL patients with the second independent cancer being either meningioma or thyroid carcinoma. None of these genes has been described to promote cancer, but *ZNF133* has been identified to be overexpressed in osteosarcoma [17]. Among the 1N patients, we detected five genes (*ZCWPW*, *SYNCRI*, *DHX3*, *DHRS4L2*, and *THSD1*) that were differentially regulated after irradiation and were located in duplicated regions. We analyzed the expression profile of *THSD1* in three independent controls (0N) and six patient-derived fibroblast cell lines (three 1N and three 2N) using qPCR and detected highly variable expression changes after radiation among controls as well as cancer patients (1N, 2N). We could not establish a clear connection between the duplication of *THSD1* and an increased expression before and after irradiation (Fig. 4).

**Fig. 5:**
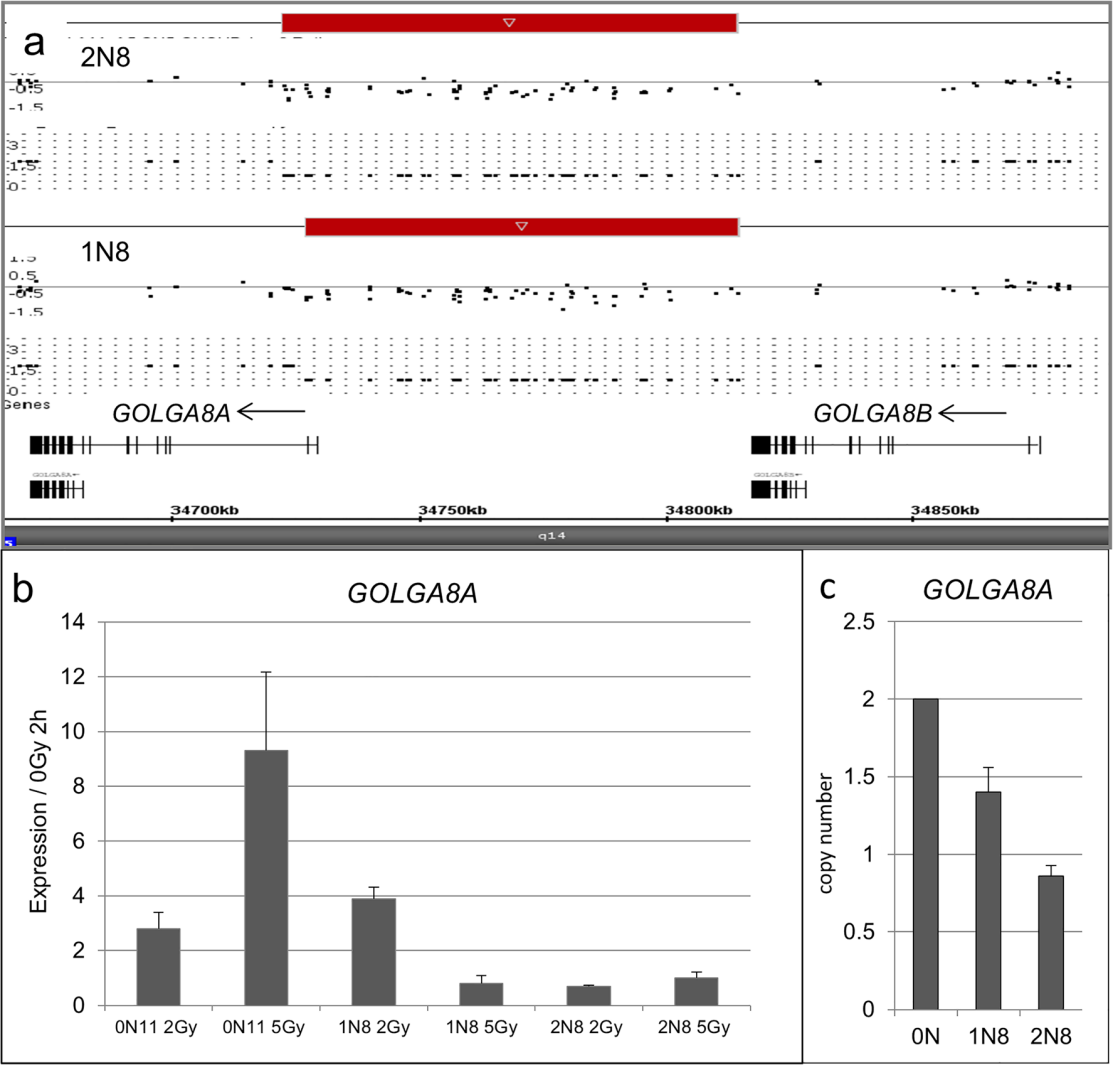
Detail analysis of a deletion in Chr.15q14 of two matched patients. Genomic deletion in 15q14, including the *GOLGAG8A* gene (**a**). RNA expression analysis using qPCR in 1N8, 2N8, and corresponding 0N11 patients. The analysis was performed with irradiated (2, 5 Gy) and control (0 Gy) cells at 2 h post-treatment (**b**). qPCR copy number analysis of this region reveals heterozygous deletions of the DNA fragment in a heterozygous state (**c**)

Other than the previously mentioned genes, we also detected radiation-sensitive genes within common CNVs. The copy number of the *DUSP22* gene is highly variable among individuals and, surprisingly, this gene changes its expression upon radiation treatment, probably contributing to individual response upon therapy. To make a classifying rating, we created a term called aberration frequency, which estimates a value for the incidence of a given aberration in a given cohort. Some alterations in the genome such as *FAM86B1* or *GOLGA8A* and duplication in the intron of *PTPRN2* were up to three times more frequent in our cancer cases than in controls. A compilation of the results is given in Online Resource 1, Table X-ray response. The highest response upon radiation (fold change) was calculated for *GOLGA8A*. The deletion occurs at the 5′ end of the retained intron transcript variant 2 non-coding RNA. qPCR examination of the copy number status in matched 1N8/0N11 and 2N8/0N11 samples revealed a loss for the 1N8 case, whereas in 2N8, the loss was heterozygous. We analyzed the expression of *GOLGA8A* before and after radiation in the corresponding matching samples. The 0N11 control shows an increase of expression after 2 h proportional to the radiation intensity, whereas the cancer patient samples 1N8 and 2N8 show a diminished response after irradiation (Fig. 5).

### Methylation analysis of duplicated genes

**Fig. 6:**
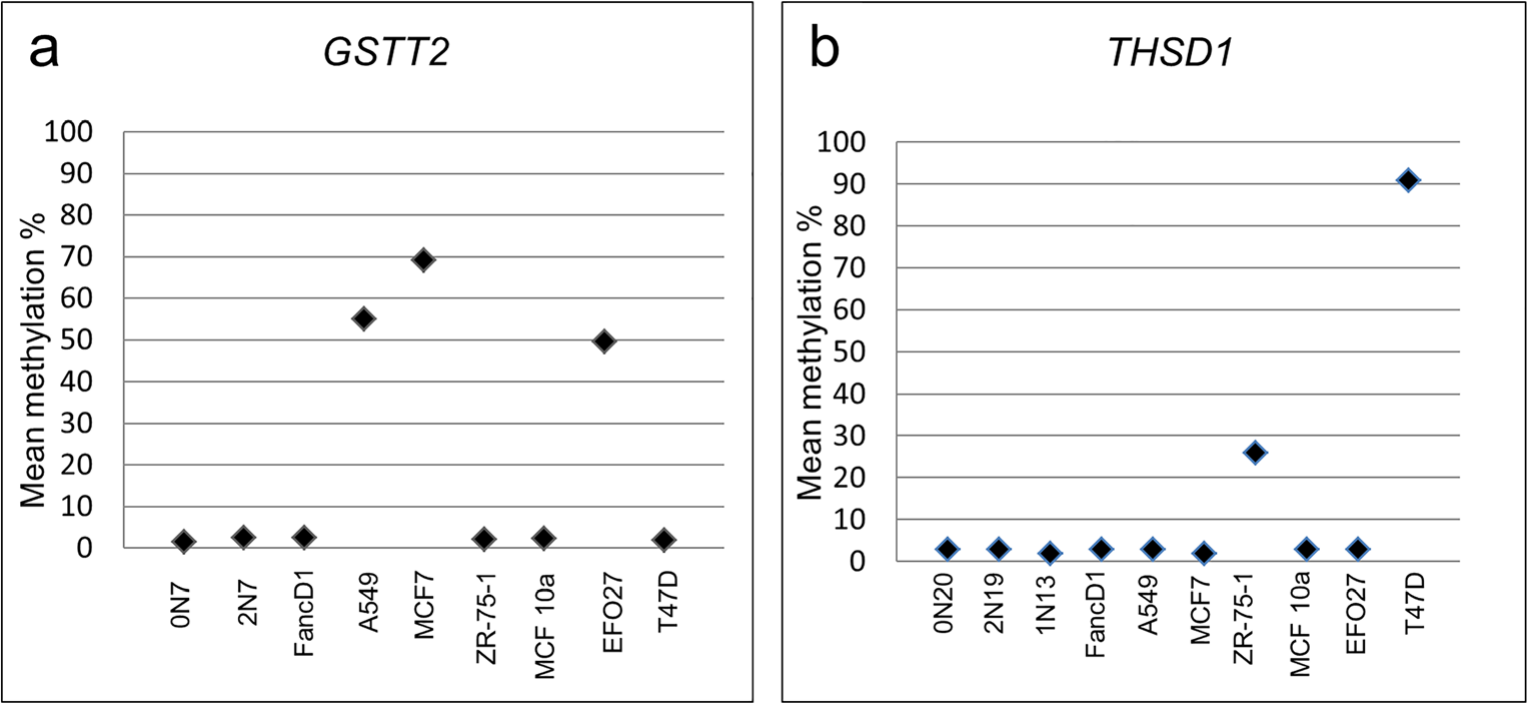
Methylation analysis of *THSD1* and *GSTT2* genes. Depicted are the mean methylation values of six CpGs for *GSTT2* and ten CpGs for *THSD1*. The tumor cell lines ZR-75-1 and T47D display hypermethylation of *THSD1* in contrast to 0N20, 2N19, 1N13, and other samples (**a**). The A549, MCF7, and EFO27 tumor cell lines show hypermethylation of *GSTT2* compared with 0N7, 2N7, and the remaining samples (**b**)

Gene expression is also modulated by methylation. In a previous study, we did not find global methylation changes in normal fetal fibroblasts 1–72 h after irradiation, neither in genic (promoters, 5′ UTRs, first exons, gene bodies, and 3′ UTRs) nor in intergenic regions [18]. To analyze the possibility of methylation changes upon altered DNA content in fibroblasts of our cancer patients, we chose two genes that presented with a CNV and were also differentially methylated in several cancer cell lines. *GSTT2* is deleted in patient 2N7 (arr[hg19] 22q11.23(24,283,003-24,330,206)x1) and in 58 participants of the control GHS collective. In comparison with the hypermethylation of the *GSTT2* CpG island, consisting of six CpGs, in the A549, MCF7, and EFO27 cancer cell lines, the patient’s sample was hypomethylated similar to the matched control sample 0N7 and the FancD1 fibroblast line. The analysis of the duplicated *THSD1* gene promoter with ten CpGs showed similar results to the *GSTT2* gene. In the case 1N13 and the matched samples 2N19 and 0N20, the values matched the values of normative samples in contrast to the hypermethylation in the two cancer cell lines ZR-75-1 and T47D **(**Fig. 6**)**.

## Discussion

### Copy number variation in cancer patients

In this study, we focused on rare CNVs, which may have an impact on cancer predisposition and recurrent cancer incidents in sporadic, non-familial, non-syndromic childhood cancer cases. There have been some studies on rare copy number aberrations (CNA) in hereditary cancer predisposition syndromes [19–22], whereas studies in sporadic cancer cases are sparse, or the cancer family history is not stated [10, 23, 24]. The possibility of CNVs occurring due to radiotherapy cannot be excluded but is unlikely because the fibroblasts in our study were usually obtained several years after the last treatment and the number of aberrant cells decreases over time and after 13 months post-therapy, normal karyotypes are prevalent [9]. The findings of acquired CNVs in leukemia patients from other studies [25, 26] did not match with the detected CNVs in our study and make them unlikely to be a secondary event. In addition, none of the CNVs in our cohort matched the reported de novo induced CNVs in clonal descendants of irradiated human fibroblasts [27]. Nevertheless, pre-existing somatic mutations acquired prior to treatment may be selected during chemotherapy/radiation and may lead to therapy-related secondary cancer [2, 28]. The aim, therefore, was to detect CNVs that could harbor genes that function as modifiers of cancer risk rather than being innocuous [29]. Since six of our patients received allogeneic bone marrow transplants, their blood DNA would represent the donor’s profile. We, therefore, did not use EBV-transformed lymphoblasts, but primary fibroblasts, which constitute a homogenous cell population with intact cell cycle and DNA repair checkpoints. To date, few childhood cancer-predisposing mutations are known [30]. The patients of our collective showed no germline mutations in high-penetrant cancer predisposition genes like *TP53* or *BRCA1/BRCA2.* None of the patients classified for a genetic cancer (predisposition) syndrome.

In our study, 75% of the former cancer patients displayed unique CNVs and very few were shared between the 1N and 2N group. Only one gene (*ABCC6*) was partially duplicated in two patients and a similar duplication was reported in an adult cancer patient by Villacis et al. [31]. A deletion of the *LINC01473* gene present in a 2N patient was reported to be deleted in a childhood cancer patient by Krepischi [23]. In 2N patients, we saw aberrations, e.g., in Chr.16p13.11, that harbor at least four genes (*ABCC1*, *FOPNL*, *MYH11*, *KIAA0430*) involved in cancer development or that are present in rare CNVs in cancer patients. Another duplicated region, 9p13.3p13.2, includes the genes *MELK*, *RNF38*, and *GNE*. The *MELK* gene is involved in cell proliferation and apoptosis whereas multiple losses of *RNF38* were detected in CML samples. Another gene that is duplicated in this region is *GNE*, which has been reported to be overexpressed in cancer (see Online Resource 1 for detailed information). Loss of it is important for the induction of apoptosis. At this point, we cannot completely exclude the possibility that the detected aberrations may have some impact on primary and secondary tumor development in 2N patients.

Similar findings were made for 1N patients. We detected a heterozygous loss of the *ST18* gene in 1N patient who suffered from leukemia, which has been previously described in two papers [32, 33]. The finding in a 1N patient of duplicated *CKAP2*, *THSD1*, and *VPS36* genes in 13q14.3 is very interesting because *CKAP2* is responsible for spindle bipolarity and chromosome stability and may represent a new factor contributing to eye tissue cancer development. As the patient was cured by surgical treatment only, the possible therapy-related changes can be excluded in this case. Altogether we would consider aberrations in *SNX14*, *SYNCRIP*, *CBR3-AS1*, and *CKAP2* as putatively responsible for tumor development or being at least an important passenger aberration in the respective 1N patients.

We verified a subset of the CNV results by qPCR and FISH analysis. Some of the CNV regions are duplicated in tandem mode and some cases may present as mosaic duplications, which conforms with the theory stated by Hu et al., [19]. As we showed in previous work, mosaic CNVs also occur in other cancer patients [34]. The absence of selective pressure might preclude the phenotypic manifestations of the minor mosaic population in a phenotypically normal individual [35], but this does not exclude the possibility of progression or change in the cell microenvironment toward cancer [36].

Some conditions might not be associated with a specific gene dosage, but rather the simple presence of a structural change at a given position in the human genome. It was stated before that intragenic regions may have an impact on cancer risk. A deleted intergenic locus may contain an enhancer which modulates breast cancer risk [37] or intergenic regions may harbor novel transcripts [38]. We, therefore, conducted a survey to find altered areas with annotated transcription binding sites in our patient collective. We included, in our study, the analysis of to date “gene free” regions and for all conspicuous areas, the presence of transcription factor binding sites, which to our knowledge, has not been done previously. These detected structural changes in cancer patient’s genomes may cause perturbation in particular pathways regardless of gene dosage. The importance of the detected “gene free” loci will be the subject of further surveys.

There are certain limitations of our study. Firstly, we were unable to collect samples from relatives to evaluate the familial status of the aberration as well as the corresponding tumor material. Also, the involvement of mutations in genes unknown to date cannot be excluded. Thirdly, there is evidence that distinct tumor classes exist, one driven by mutations and the other driven by CNVs. The question if the detected CNVs may represent driver or passenger mutations cannot be answered at this stage. Due to the chosen technique, only duplications and deletions are described in this study, while balanced structural changes remain undetected. Each patient displayed an almost unique pattern of aberrations which is consistent with the idea of the multi-causation of cancer and findings of spontaneous abnormalities in normal human fibroblasts from patients with Li-Fraumeni cancer syndrome [39] and chromosomal changes in non-cancerous breast tissue for breast carcinoma patients [40]. Interestingly, an abnormal gene expression in fibroblasts was detected in patients with Gorlin syndrome (GS), which is a hereditary disorder with tumorigenicity, caused by constitutive hyperactivity of hedgehog signaling. The hyper-activated hedgehog signaling contributes to low miR-196a-5p expression and high MAP3K1 expression in human fibroblasts and mouse cells [41]. The described CNVs in this study may consequently deregulate gene expression and important pathways. Although it was not possible to connect the occurrence of particular CNVs and 2N or 1N cancer incidence, our findings may inspire new insights in cancer pathways regulation. To our knowledge, this is the first time that such an investigation in childhood cancer survival patients prevalent in ALL and matched triplet design and additional 1000 well-chosen controls was performed. Further studies are on the way with increased patient numbers to corroborate our findings.

### Radiation sensitive genes inside copy number variations

We generated an extensive gene list, which represents the dose- and time-dependent response upon irradiation damage to define sensitive genes within CNVs. qPCR analysis and former studies with radiation-induced transcriptional responses performed on quiescent fibroblasts support our results. We detected altered regions harboring genes, which respond upon irradiation and were not described to date as radiation-sensitive, but some of the detected genes were described in cancer. One gene (*ZNF133*), which responded upon irradiation, was detected in a patient of the 2N collective and has been identified as being overexpressed in osteosarcoma [17]. More genes described in cancer were detected in 1N patients. The gene *ZCWPW2* is a histone-modifying enzyme [42] and was downregulated after radiation in controls. The gene locus was found to be duplicated in a patient who suffered from Hodgkin’s lymphoma. *SYNCRIP*, an RNA-binding protein that controls the myeloid leukemia stem cell program [43], was found to be overexpressed after radiation and was duplicated in a patient with sarcoma. *DHX30*, which was found to be frequently mutated in childhood AML [44], was duplicated in a patient with ALL. The *THSD1* gene is often mutated in cancer [45] and was found to be duplicated in a patient with unilateral retinoblastoma (1N13), lacking an *RB1* mutation. None of the findings may explain the proneness to secondary cancers in 2N participants of this study. One of the most likely reasons for this result is the limited number of patients. Additional studies by analyzing larger cohorts may thus uncover more responsible gene sites. We show exemplarily that gene expression may depend upon copy number alterations but there are also exceptions. *THSD1* proved to be expressed in some individuals after irradiation on a copy number independent status. This is not unusual, because individual gene expression response after genotoxic agents in fibroblasts has already been described. Such findings complicate future studies and make intensive studies on RNA and protein level necessary. In some frequency, genomic alterations in gene copy numbers were seen also in controls, which may indicate a certain individual plasticity upon damage. Genes like *DUSP22*, which is deleted in some cases of cutaneous anaplastic large T cell lymphomas, may have more than one physiological substrate and the regulation of specific signaling cascades by this enzyme may be cell-type and context-specific. Recently, *DUSP22* was described to contribute to inflammatory bowel disease [46]. Surprisingly, its copy number varies in fibroblasts in 0N cases as well as in our patients. Another gene that was affected in controls and more frequently in our patients may play a role in tumor tissue. Significant biallelic deletions of *GOLGA8A* have been described in gastrointestinal tumors and pancreatic ductal adenocarcinomas. The detected downregulation in our patients correlates with the deletion status of the region, as shown also by Wang et al., [47].

It was shown that CNVs may be associated with aberrant methylation and have an impact on tumor prognosis [48]. Thus, to compensate for the addition or loss of genetic material, affected genes may be fine-tuned by methylation [10, 49]. In our two analyzed gene loci (*THSD1* and *GSTT2*), no aberrant methylation in patients was detected in contrast to hypermethylation in the analyzed tumor cell lines. Nevertheless, additional methylation surveys should be conducted in further studies.

To our knowledge, this is the first study that uses primary fibroblasts of childhood sporadic cancer cases. In conclusion, although we did not detect a consistent overall candidate gene, we describe potential vulnerable sites or rare CNVs in our collective, which may contribute to tumor development. Furthermore, we detected genes sensitive to radiation treatment that are transcriptionally altered by CNVs. As we detected aberrations seen also by other researchers, it is worthwhile to conduct further investigations in a larger collective and extensive study to address expression, cellular localization, putative deletion, and overexpression of genes to determine the impact of a given aberration on maintaining genome stability.

## Electronic supplementary material

ESM 1 (XLSX 68 kb)

ESM 2 (XLSX 46980 kb)

ESM 3 (PNG 455 kb)

High Resolution Image (TIF 523 kb)

ESM 4 (PNG 386 kb)

High Resolution Image (TIF 466 kb)

ESM 5 (PNG 540 kb)

High Resolution Image (TIF 633 kb)

ESM 6 (PNG 556 kb)

High Resolution Image (TIF 664 kb)

ESM 7 (PNG 589 kb)

High Resolution Image (TIF 714 kb)

ESM 8 (PNG 490 kb)

High Resolution Image (TIF 571 kb)

ESM 9 (PNG 553 kb)

High Resolution Image (TIF 655 kb)

ESM 10 (PNG 570 kb)

High Resolution Image (TIF 691 kb)

ESM 11 (PNG 496 kb)

High Resolution Image (TIF 580 kb)

ESM 12 (PNG 650 kb)

High Resolution Image (TIF 729 kb)

ESM 13 (PNG 514 kb)

High Resolution Image (TIF 595 kb)

ESM 14 (PNG 503 kb)

High Resolution Image (TIF 581 kb)

ESM 15 (PNG 527 kb)

High Resolution Image (TIF 620 kb)

ESM 16 (PNG 580 kb)

High Resolution Image (TIF 659 kb)

ESM 17 (PNG 478 kb)

High Resolution Image (TIF 515 kb)

ESM 18 (DOCX 132 kb)

## Data Availability

All data generated or analyzed during this study are included in this published article (and its Online Resources).
