# Supplementary figures and images for: Molecular karyotyping and gene expression analysis in childhood cancer patients
Source: J Mol Med (Berl). 2020 Jun 23;98(8):1107–23. doi: 10.1007/s00109-020-01937-4 (PMC7769790; doi:10.1007/s00109-020-01937-4)

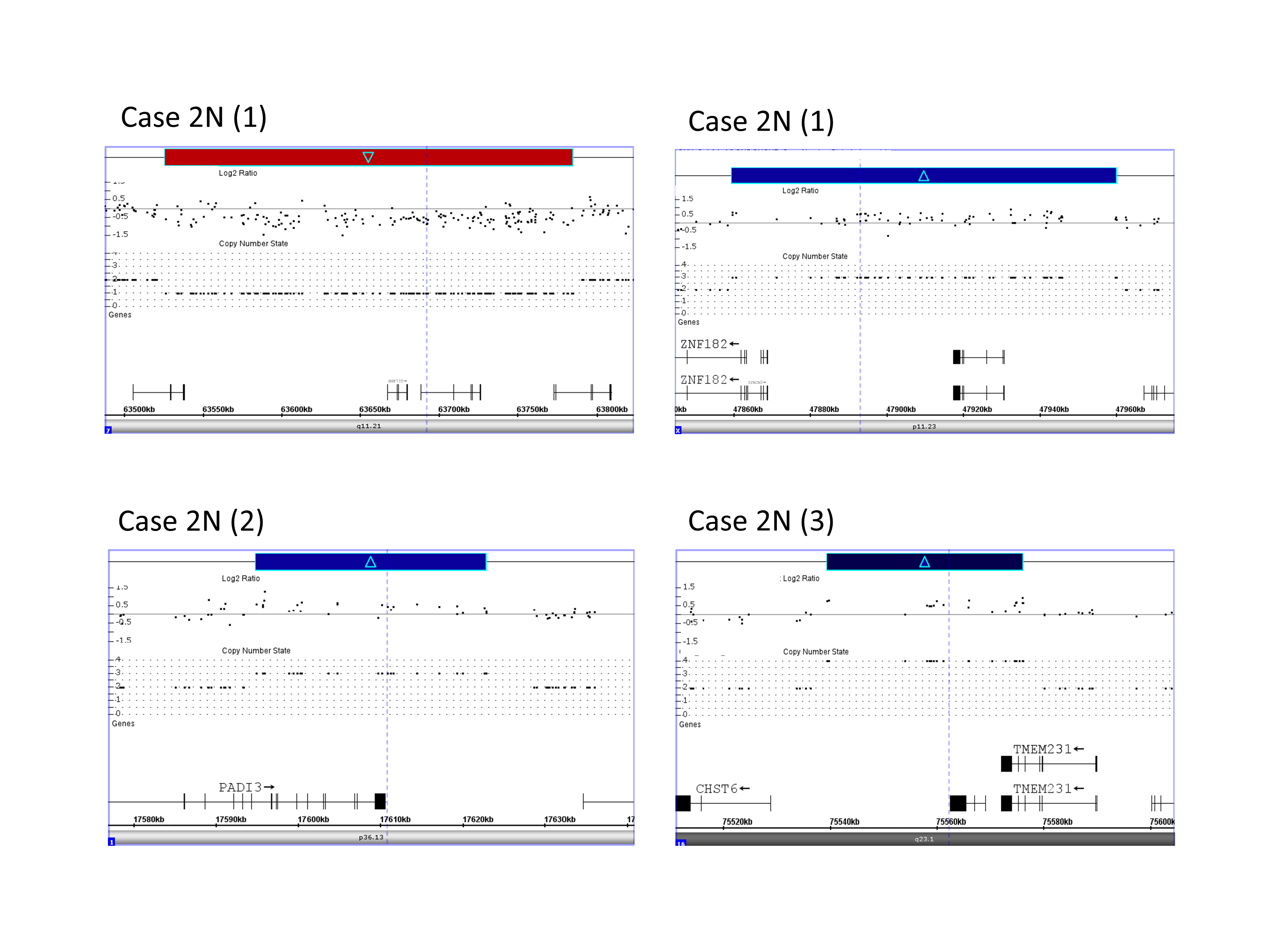

Supplement: Supplementary file 3 — (PNG 455 kb) [file 109_2020_1937_Fig7_ESM.png]

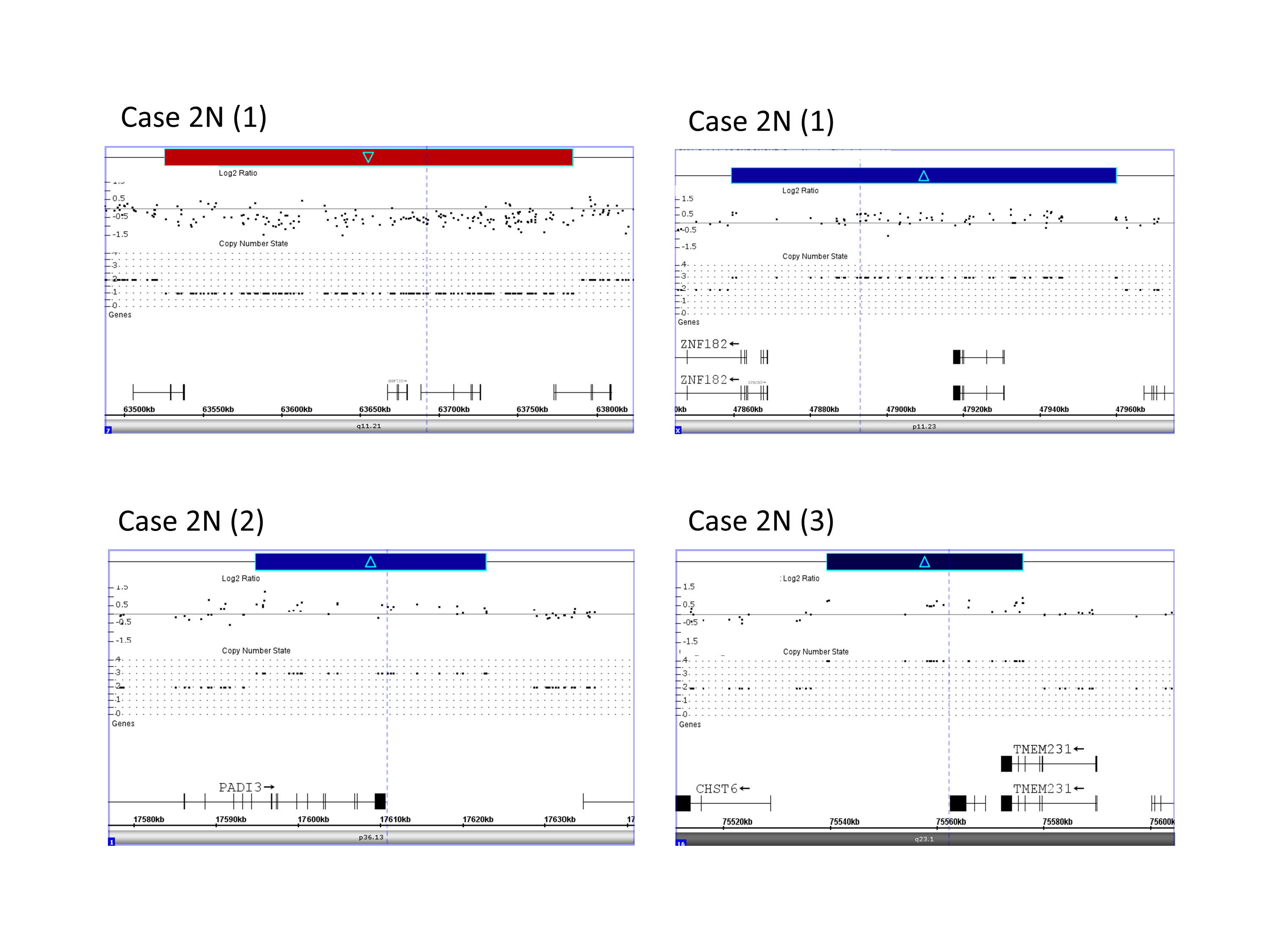

Supplement: Supplementary file 4 — High Resolution Image (TIF 523 kb) [file 109_2020_1937_MOESM3_ESM.tif]

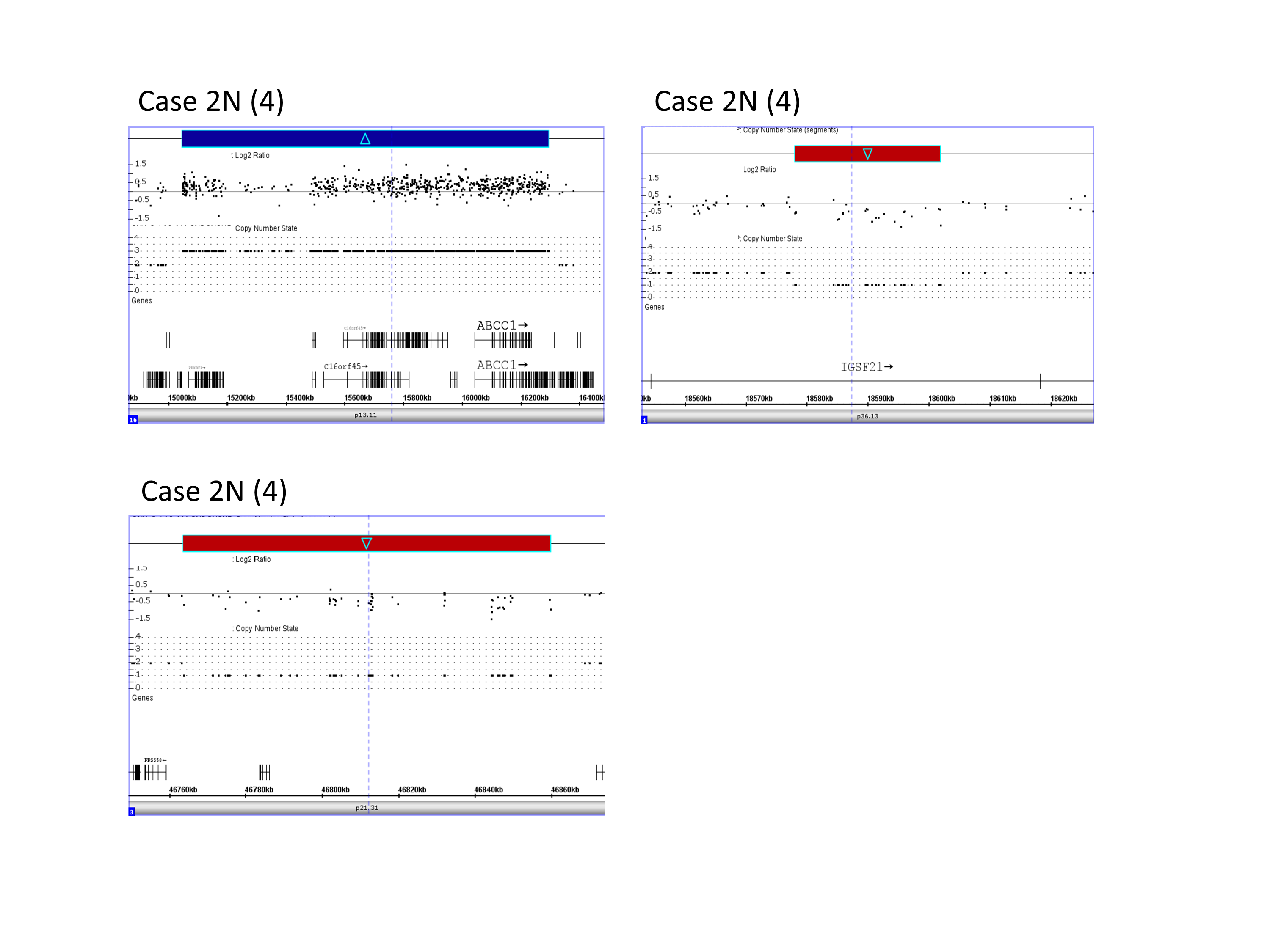

Supplement: Supplementary file 5 — (PNG 386 kb) [file 109_2020_1937_Fig8_ESM.png]

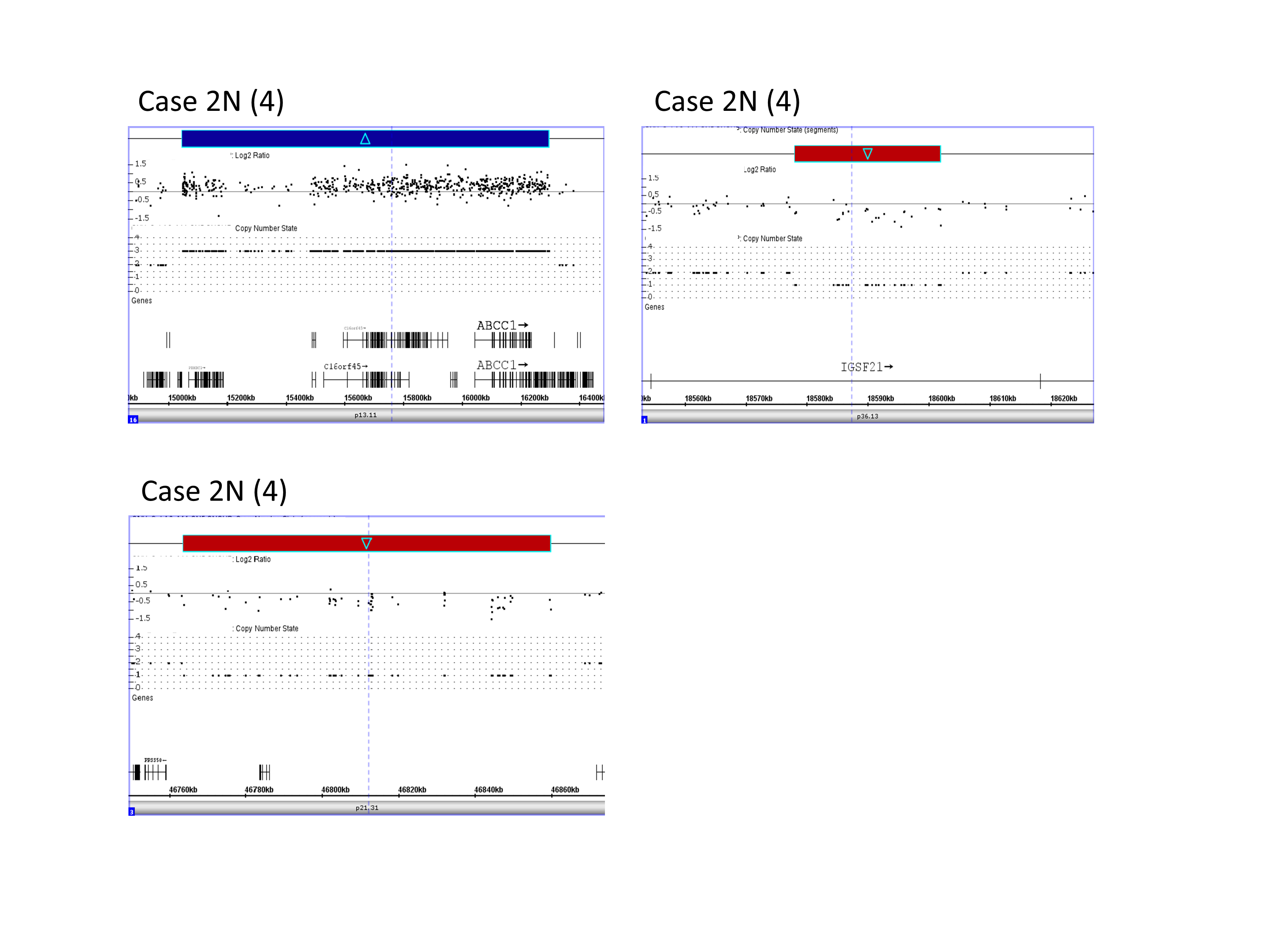

Supplement: Supplementary file 6 — High Resolution Image (TIF 466 kb) [file 109_2020_1937_MOESM4_ESM.tif]

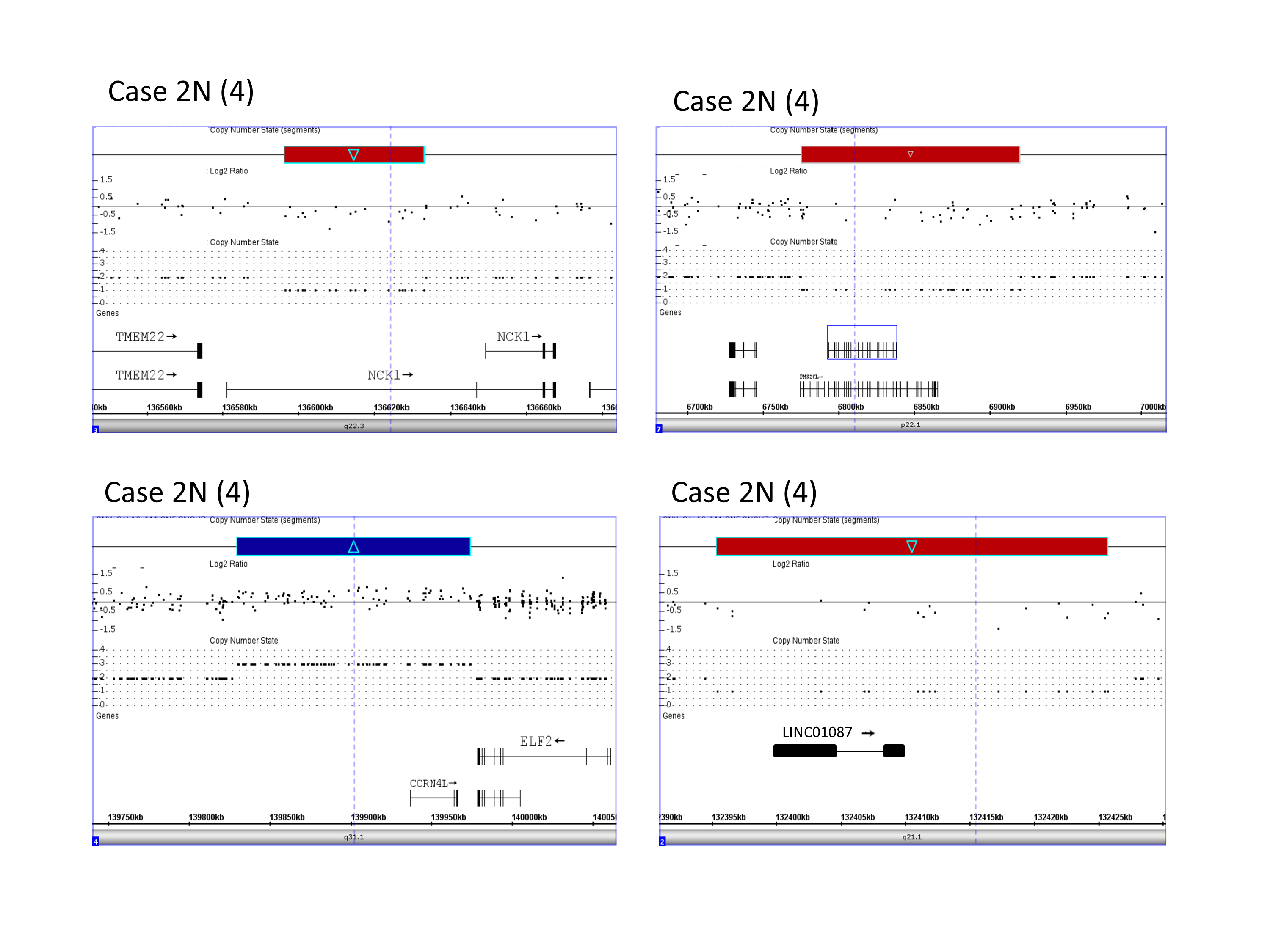

Supplement: Supplementary file 7 — (PNG 540 kb) [file 109_2020_1937_Fig9_ESM.png]

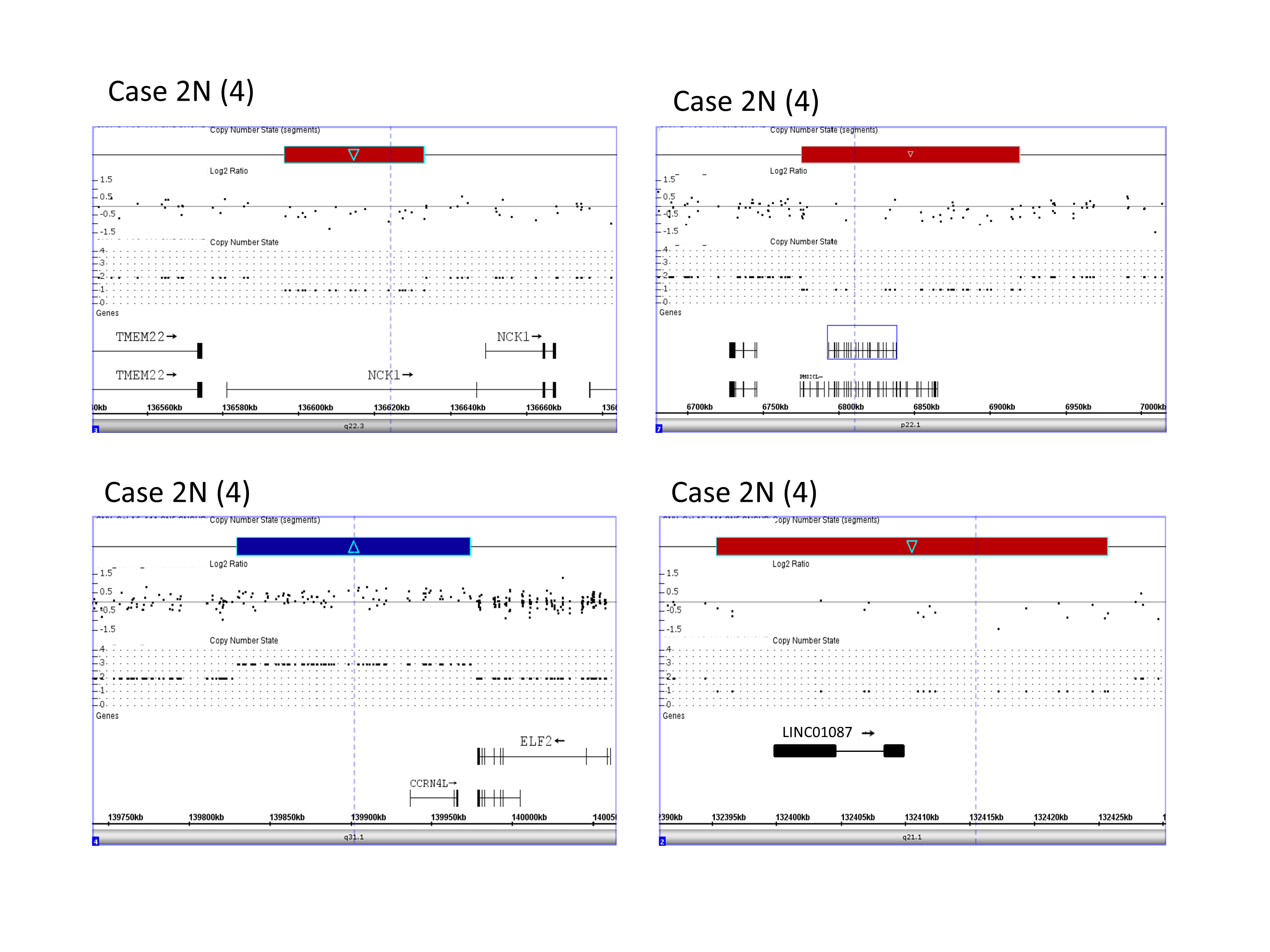

Supplement: Supplementary file 8 — High Resolution Image (TIF 633 kb) [file 109_2020_1937_MOESM5_ESM.tif]

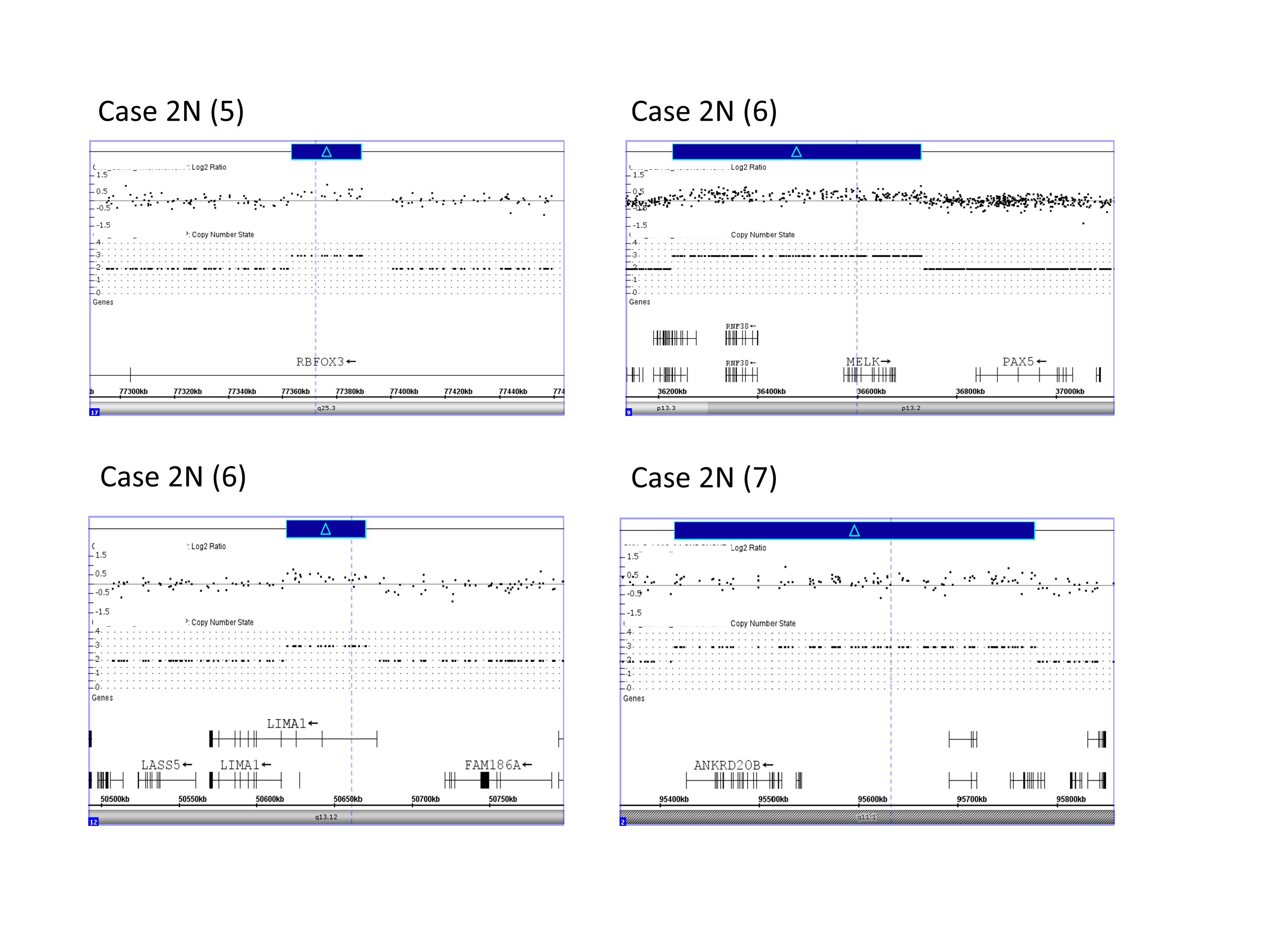

Supplement: Supplementary file 9 — (PNG 556 kb) [file 109_2020_1937_Fig10_ESM.png]

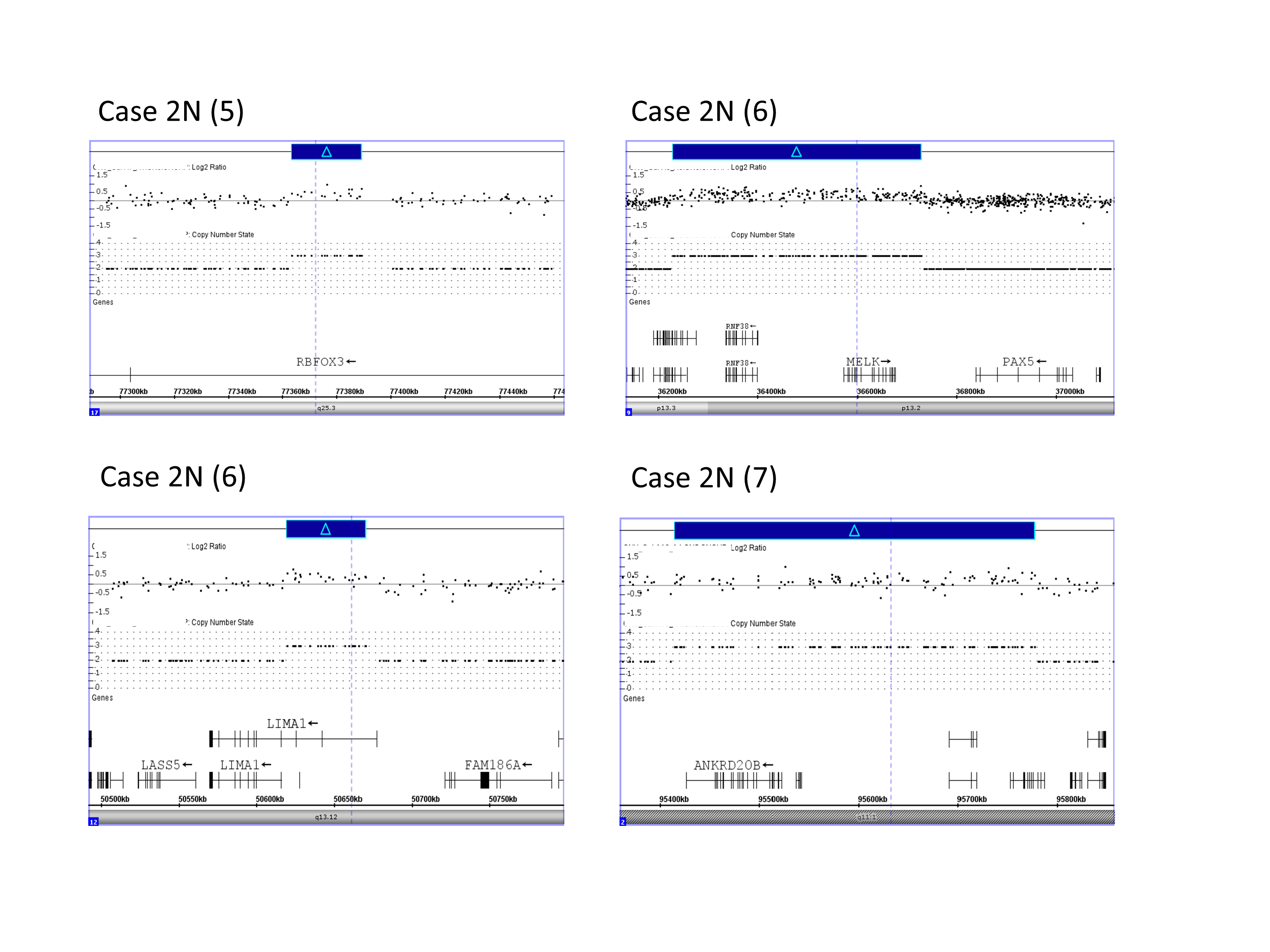

Supplement: Supplementary file 10 — High Resolution Image (TIF 664 kb) [file 109_2020_1937_MOESM6_ESM.tif]

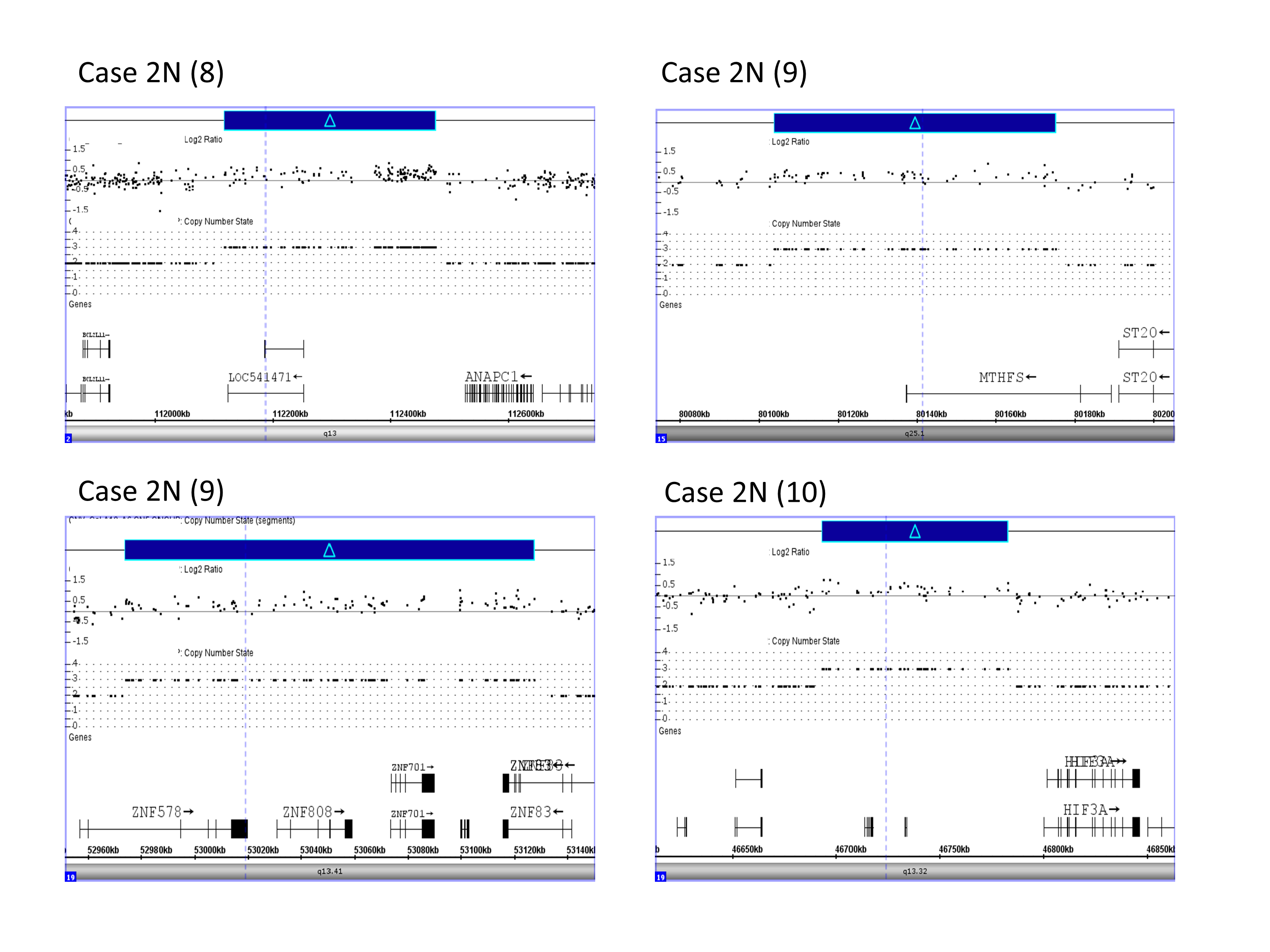

Supplement: Supplementary file 11 — (PNG 589 kb) [file 109_2020_1937_Fig11_ESM.png]

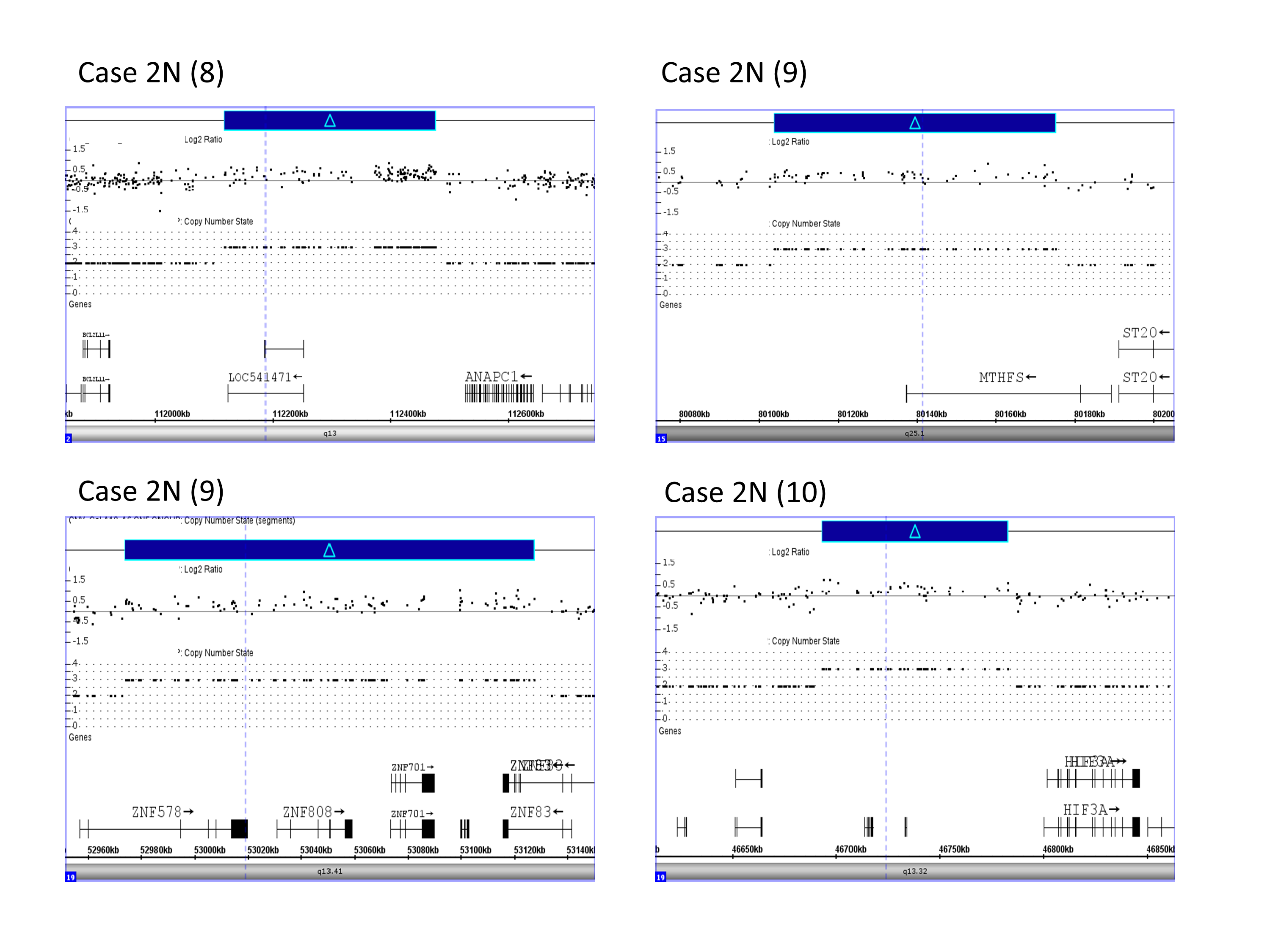

Supplement: Supplementary file 12 — High Resolution Image (TIF 714 kb) [file 109_2020_1937_MOESM7_ESM.tif]

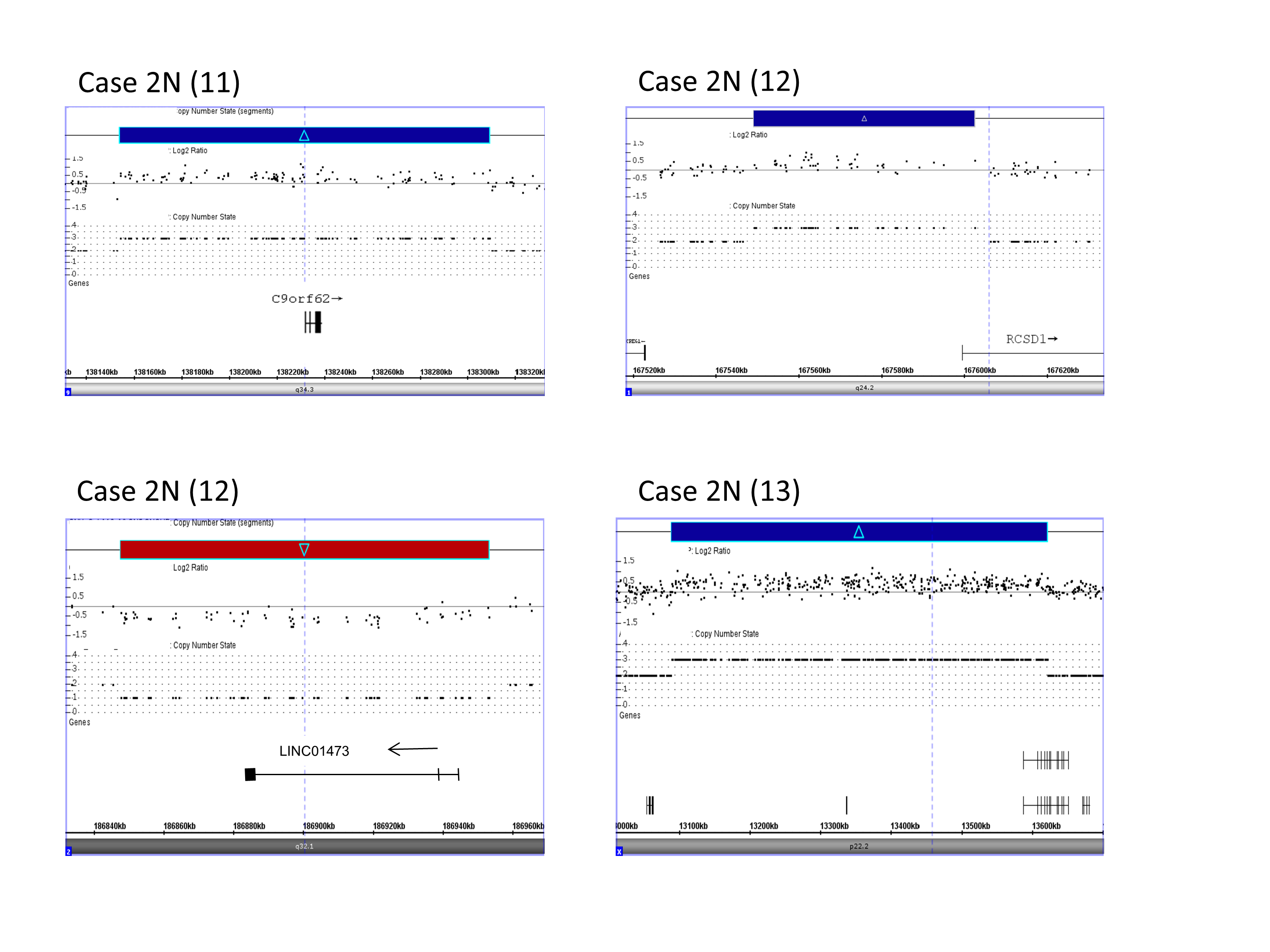

Supplement: Supplementary file 13 — (PNG 490 kb) [file 109_2020_1937_Fig12_ESM.png]

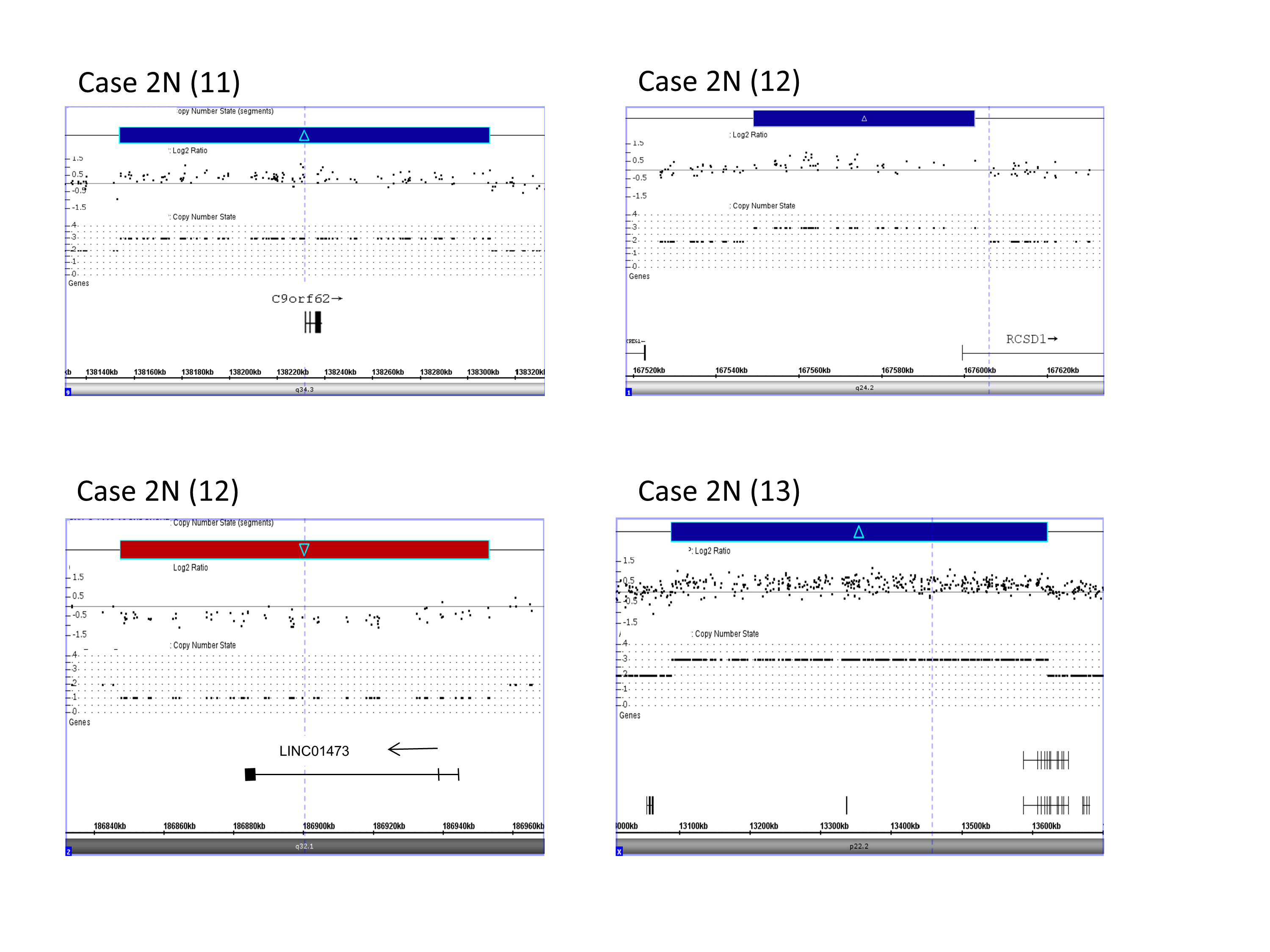

Supplement: Supplementary file 14 — High Resolution Image (TIF 571 kb) [file 109_2020_1937_MOESM8_ESM.tif]

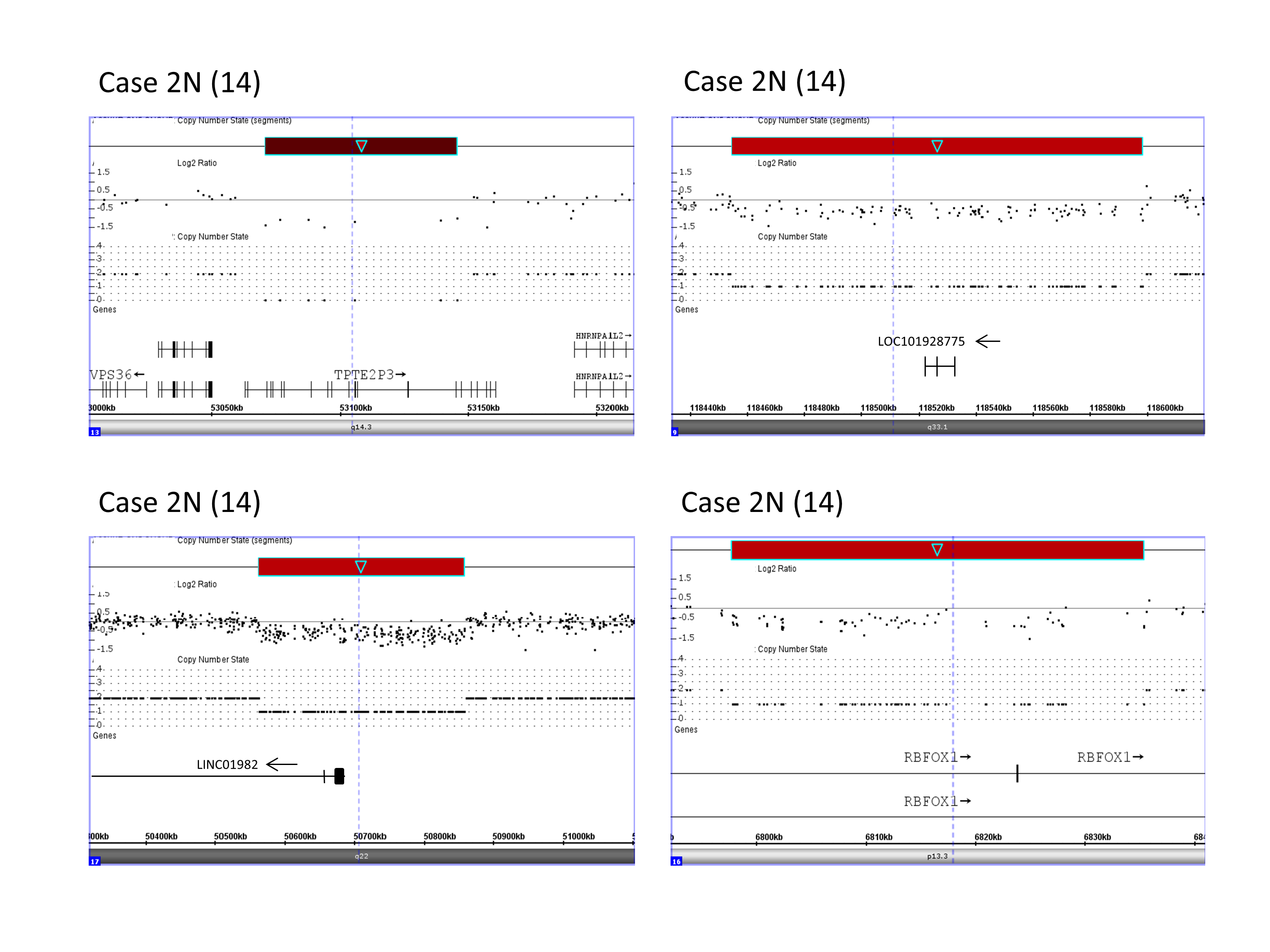

Supplement: Supplementary file 15 — (PNG 553 kb) [file 109_2020_1937_Fig13_ESM.png]

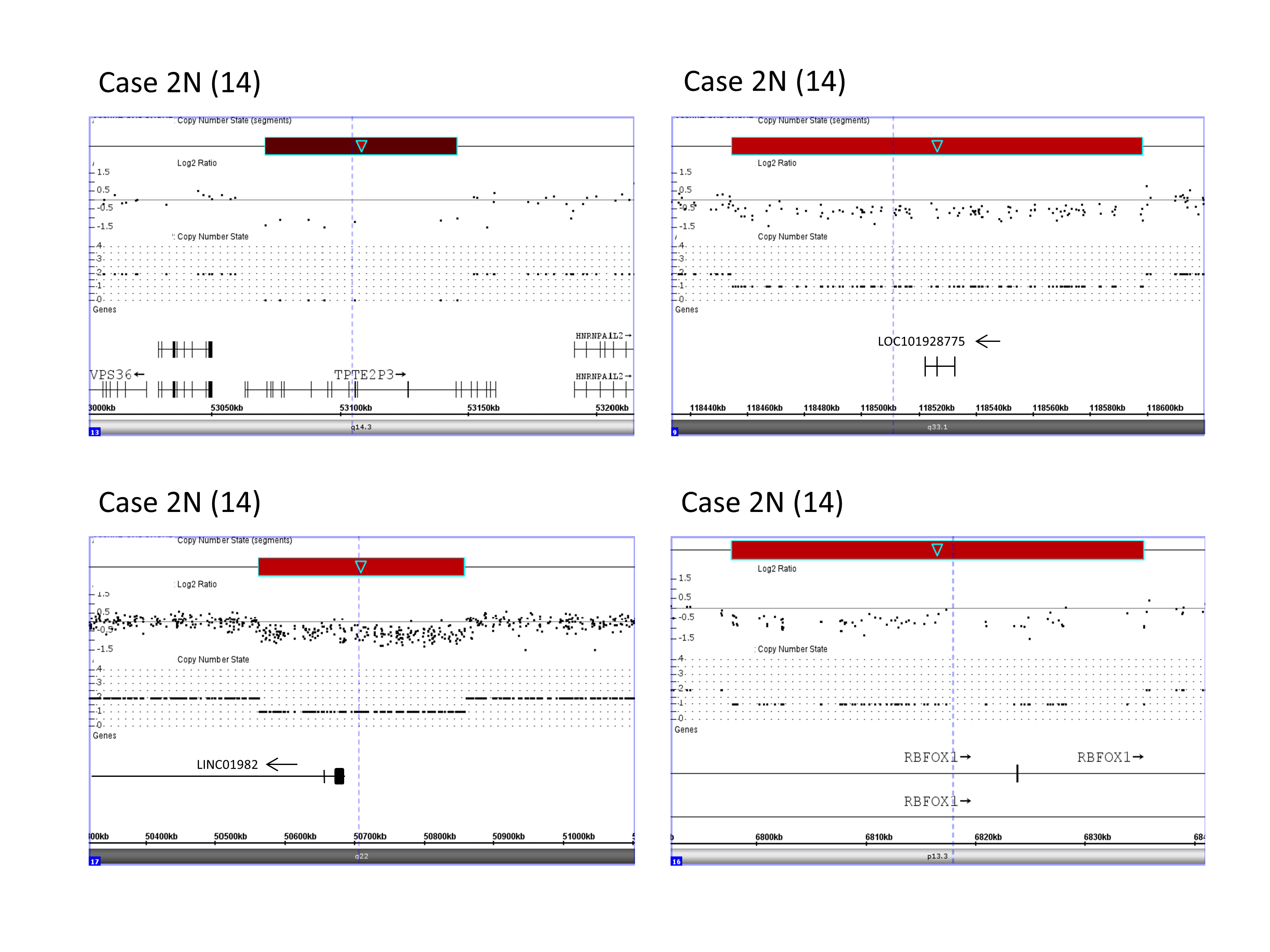

Supplement: Supplementary file 16 — High Resolution Image (TIF 655 kb) [file 109_2020_1937_MOESM9_ESM.tif]

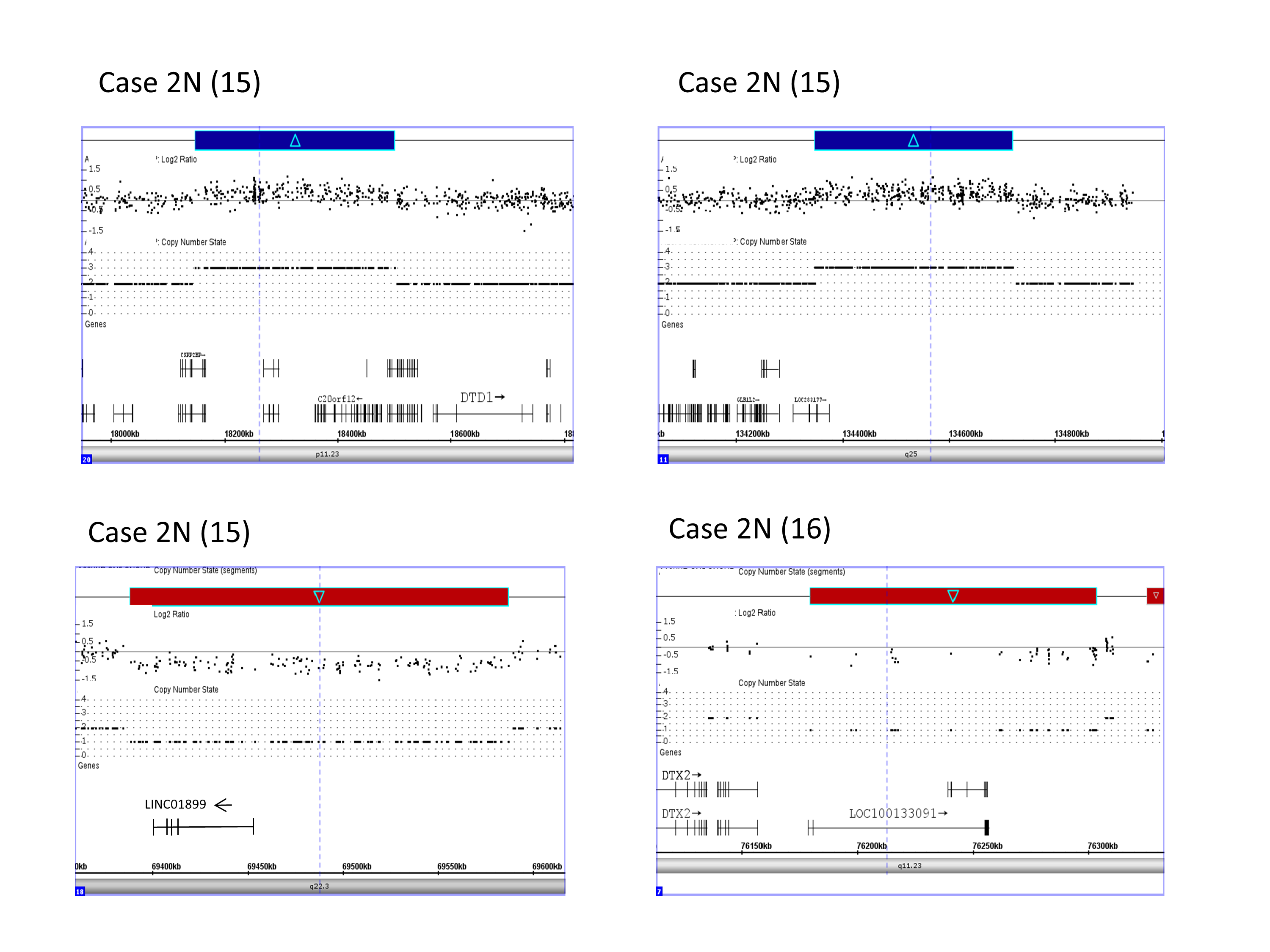

Supplement: Supplementary file 17 — (PNG 570 kb) [file 109_2020_1937_Fig14_ESM.png]

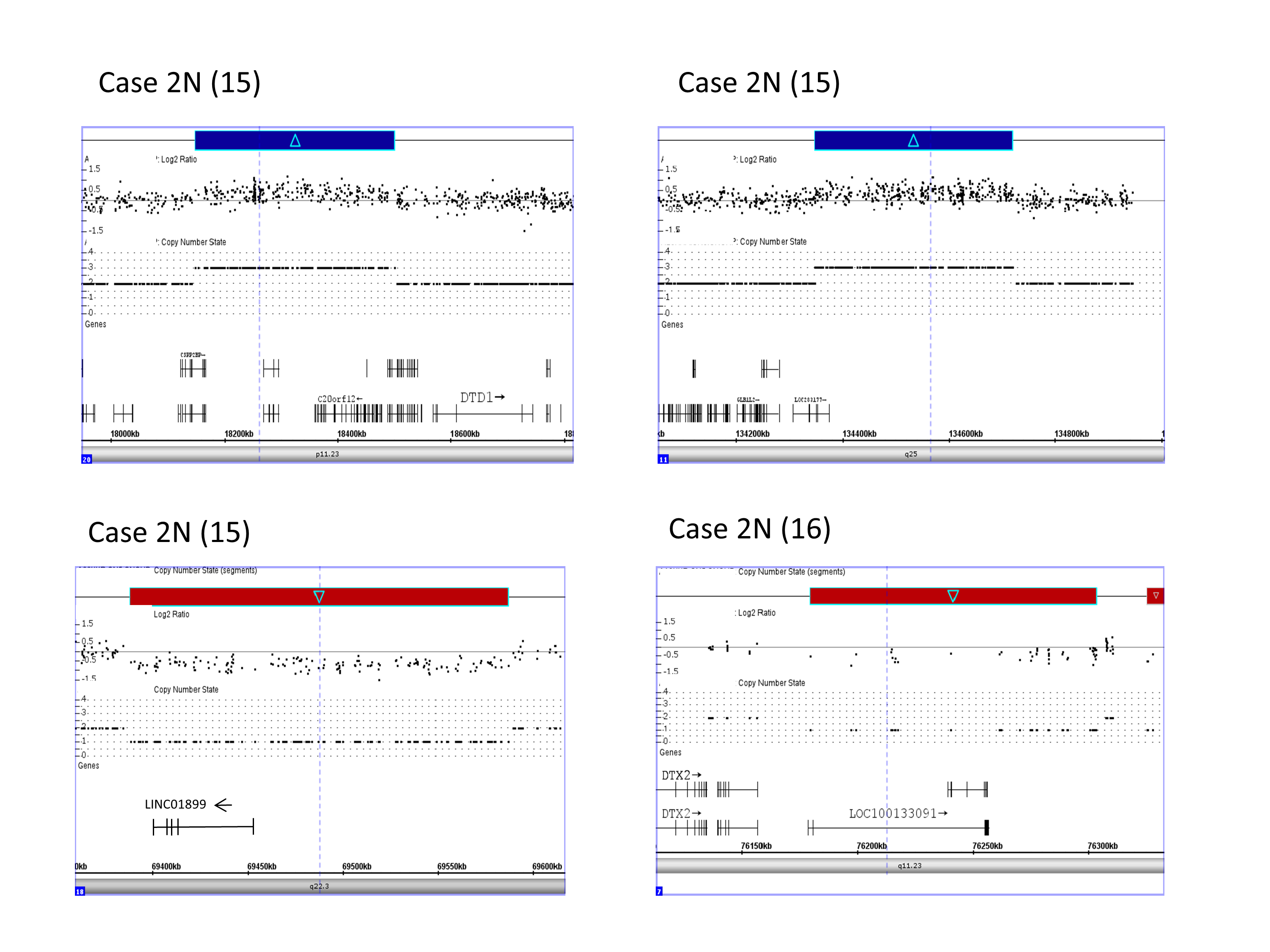

Supplement: Supplementary file 18 — High Resolution Image (TIF 691 kb) [file 109_2020_1937_MOESM10_ESM.tif]

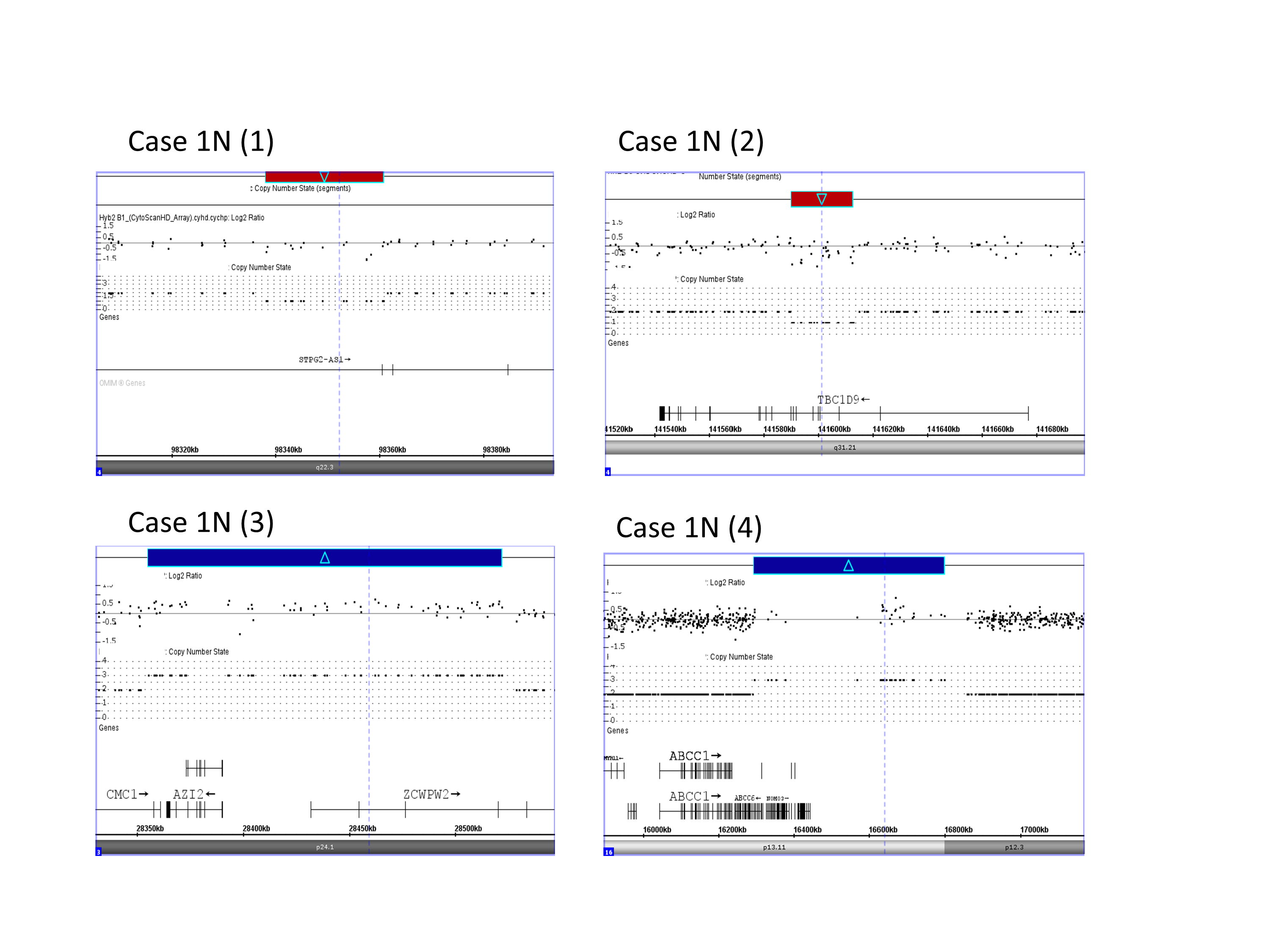

Supplement: Supplementary file 19 — (PNG 496 kb) [file 109_2020_1937_Fig15_ESM.png]

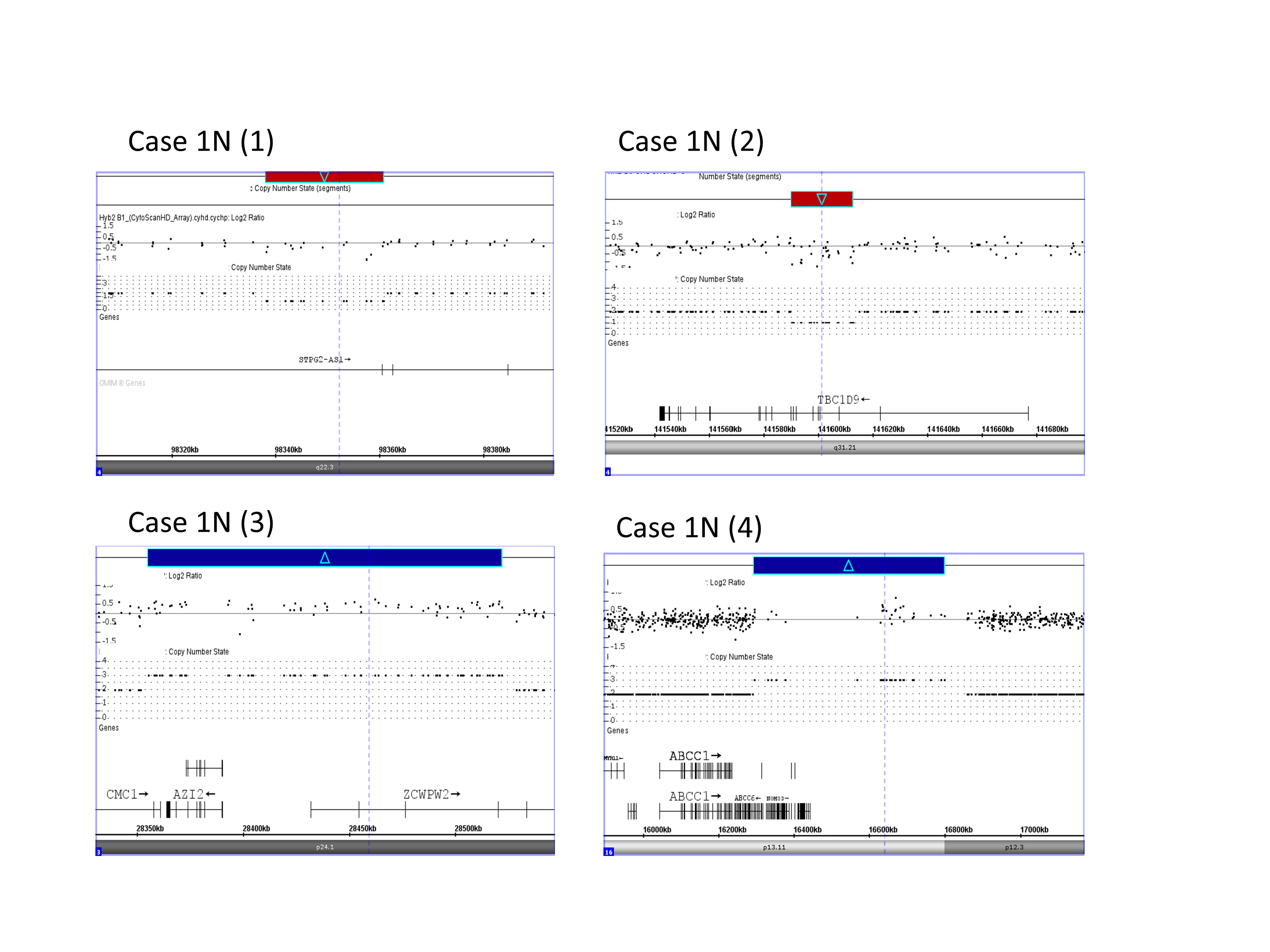

Supplement: Supplementary file 20 — High Resolution Image (TIF 580 kb) [file 109_2020_1937_MOESM11_ESM.tif]

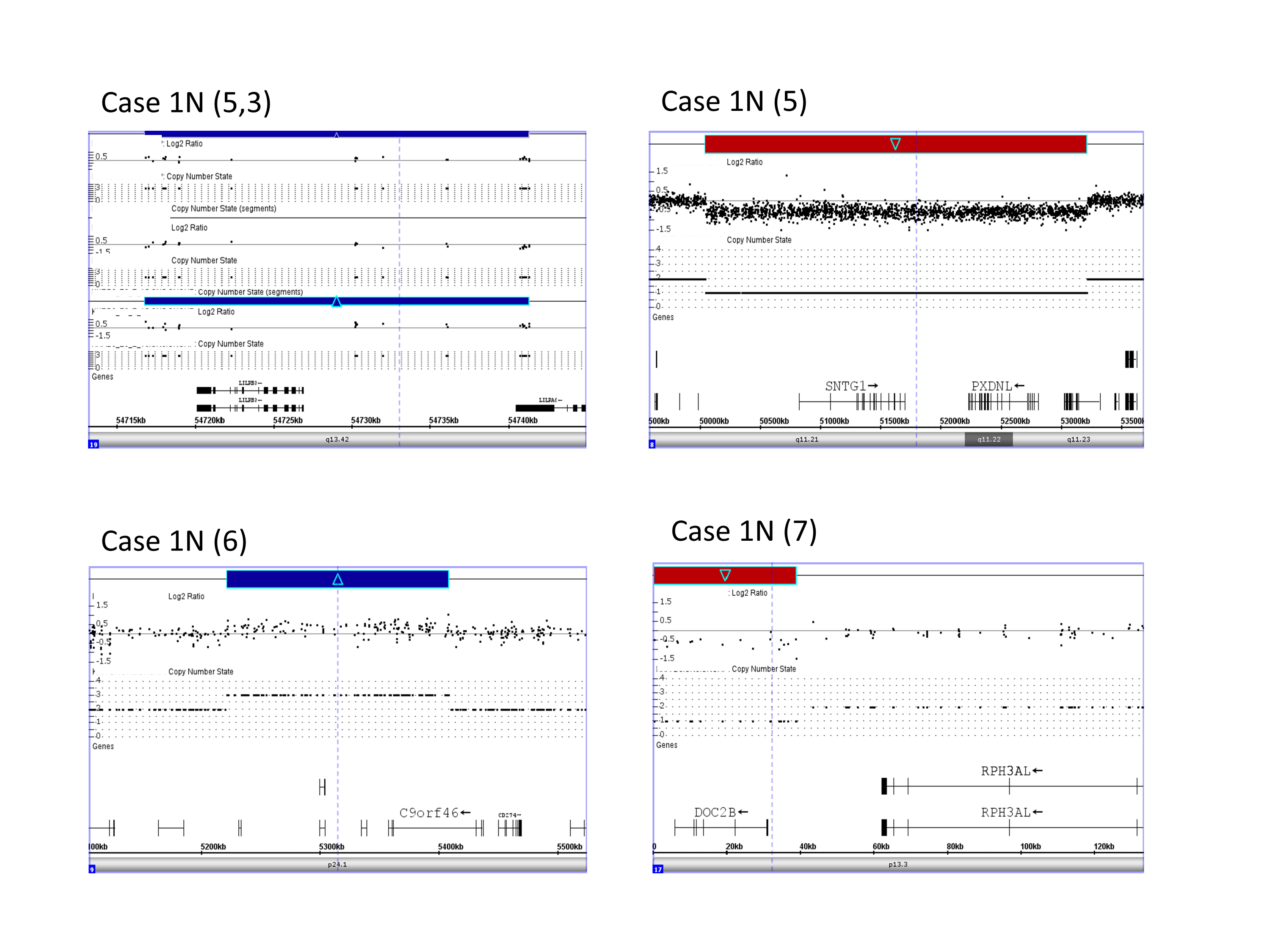

Supplement: Supplementary file 21 — (PNG 650 kb) [file 109_2020_1937_Fig16_ESM.png]

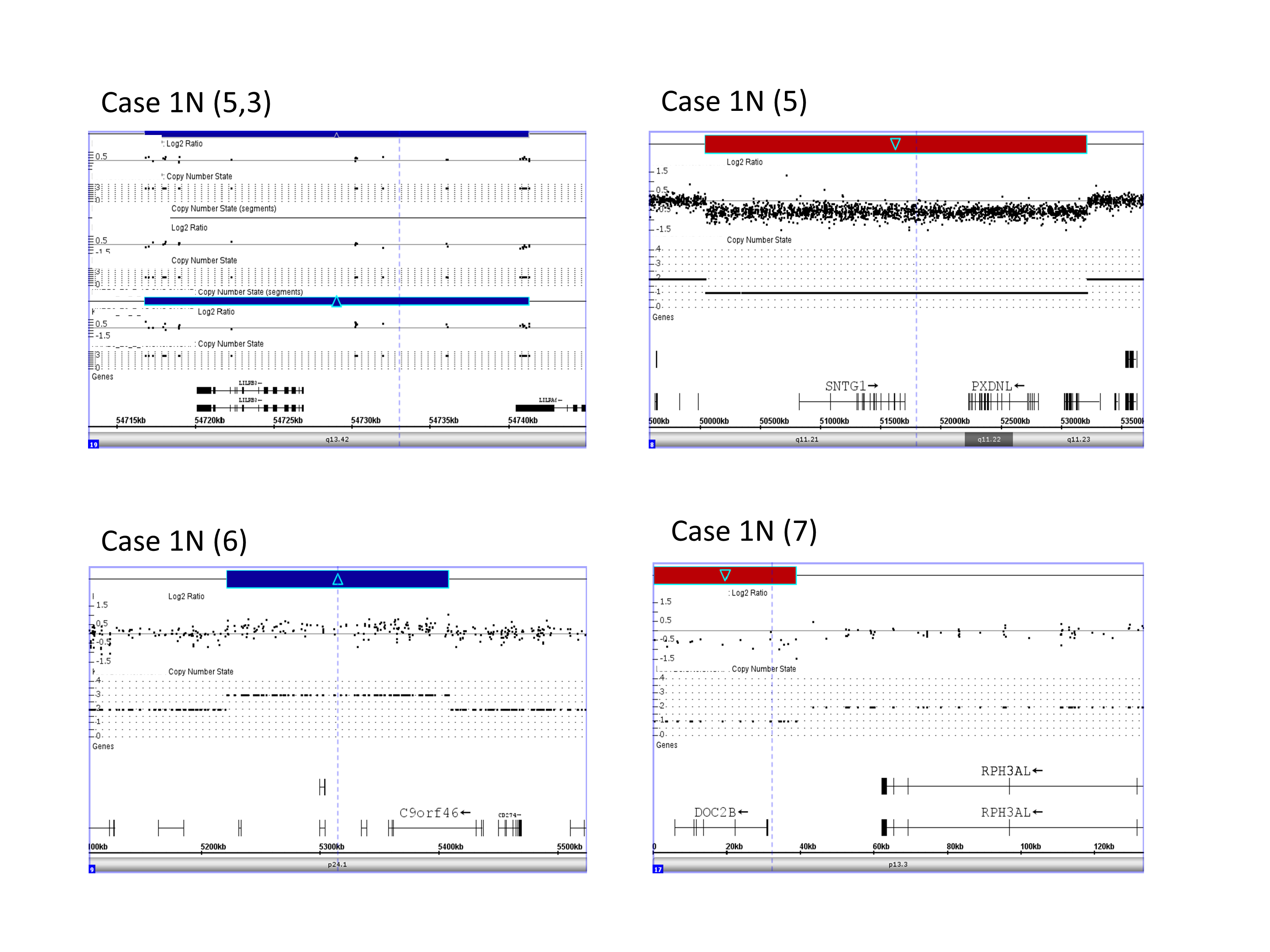

Supplement: Supplementary file 22 — High Resolution Image (TIF 729 kb) [file 109_2020_1937_MOESM12_ESM.tif]

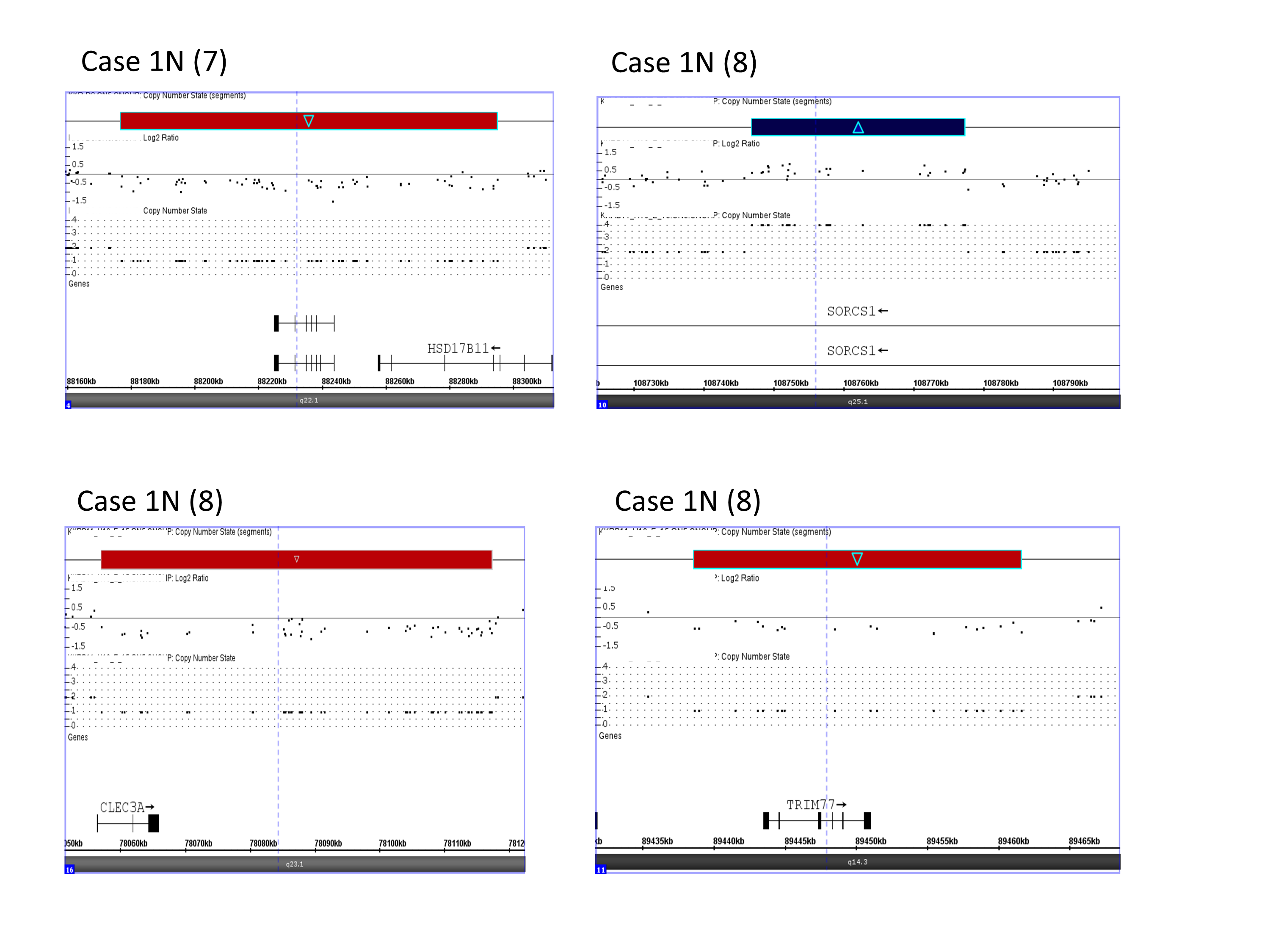

Supplement: Supplementary file 23 — (PNG 514 kb) [file 109_2020_1937_Fig17_ESM.png]

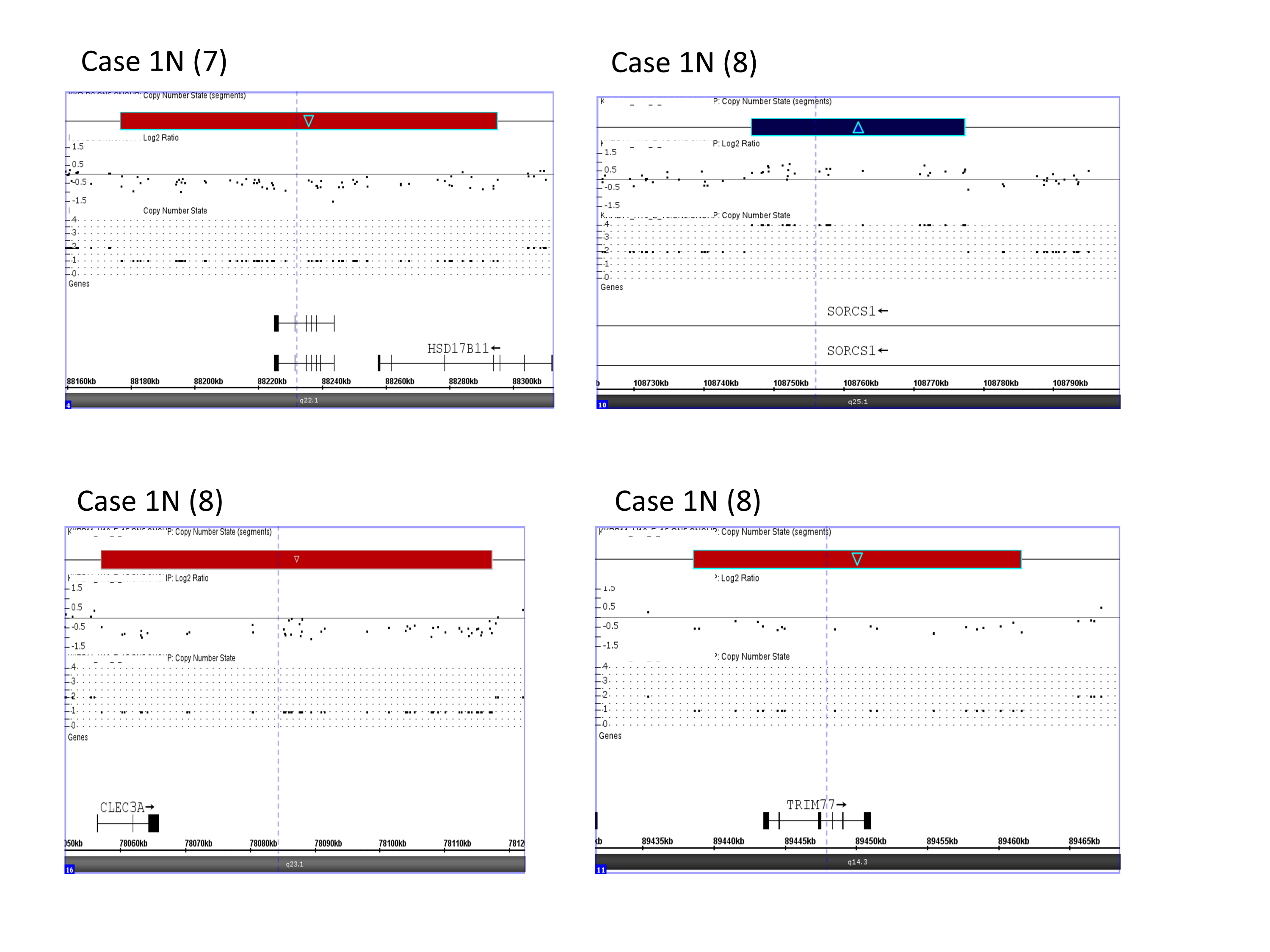

Supplement: Supplementary file 24 — High Resolution Image (TIF 595 kb) [file 109_2020_1937_MOESM13_ESM.tif]

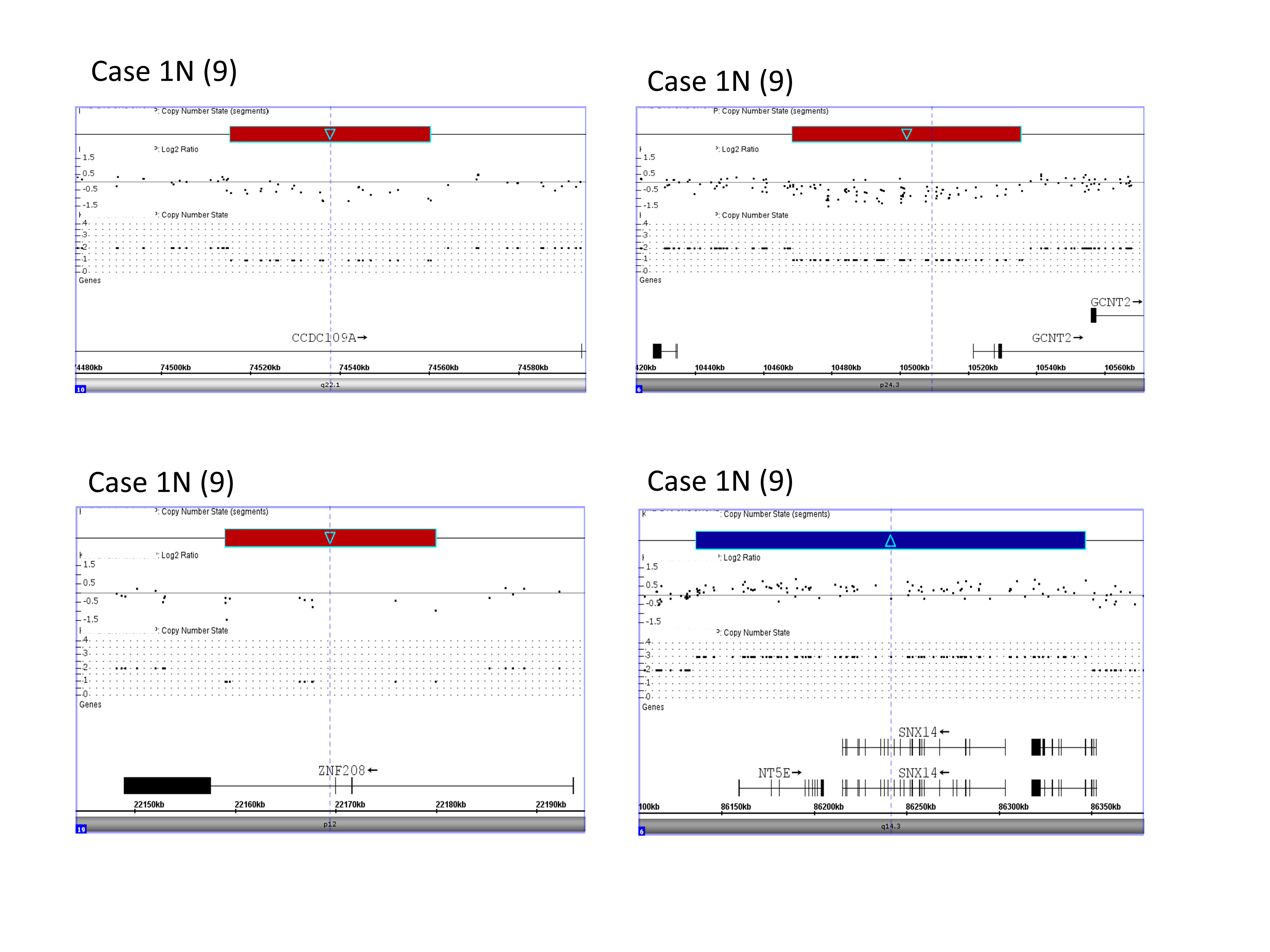

Supplement: Supplementary file 25 — (PNG 503 kb) [file 109_2020_1937_Fig18_ESM.png]

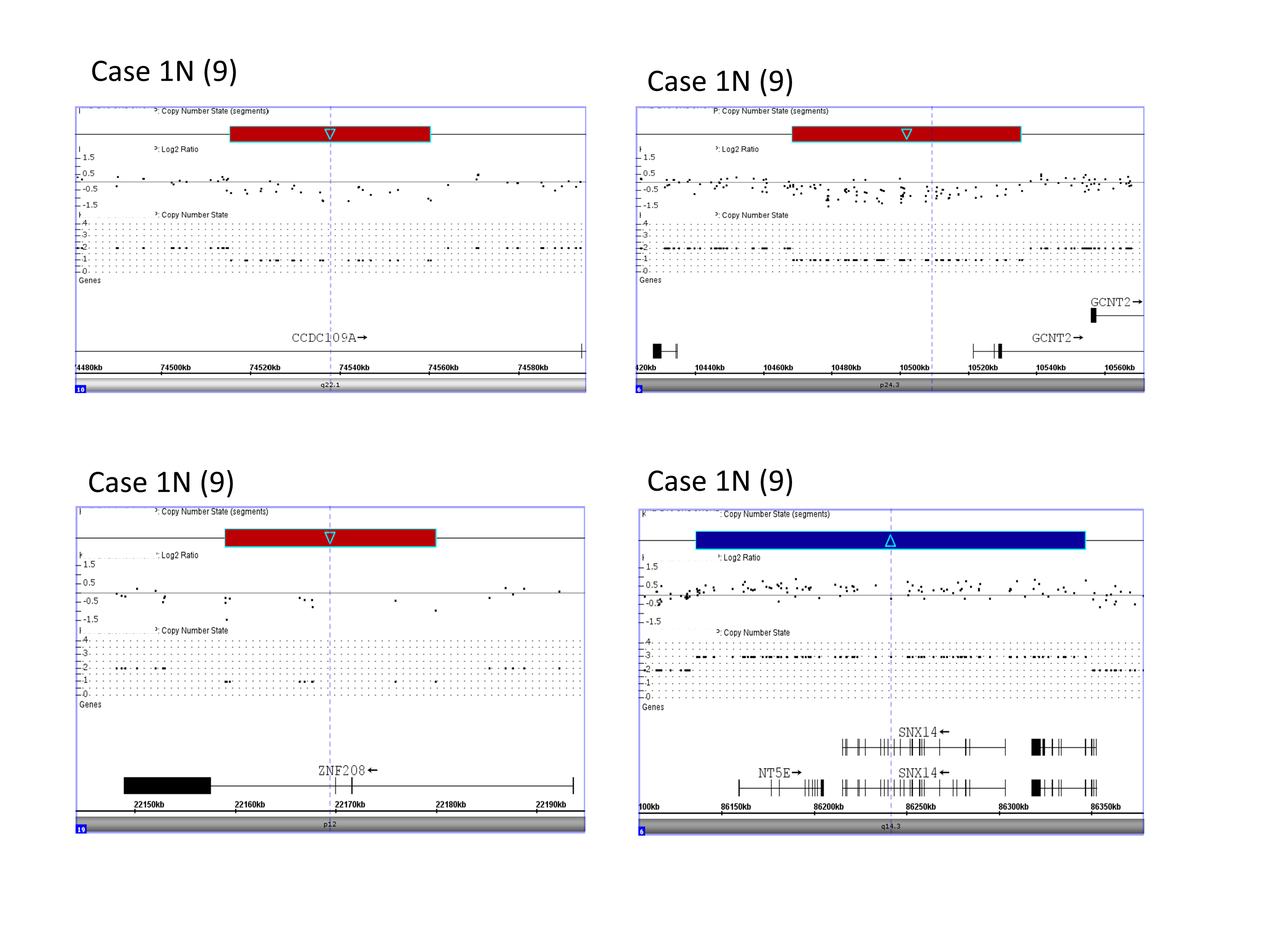

Supplement: Supplementary file 26 — High Resolution Image (TIF 581 kb) [file 109_2020_1937_MOESM14_ESM.tif]

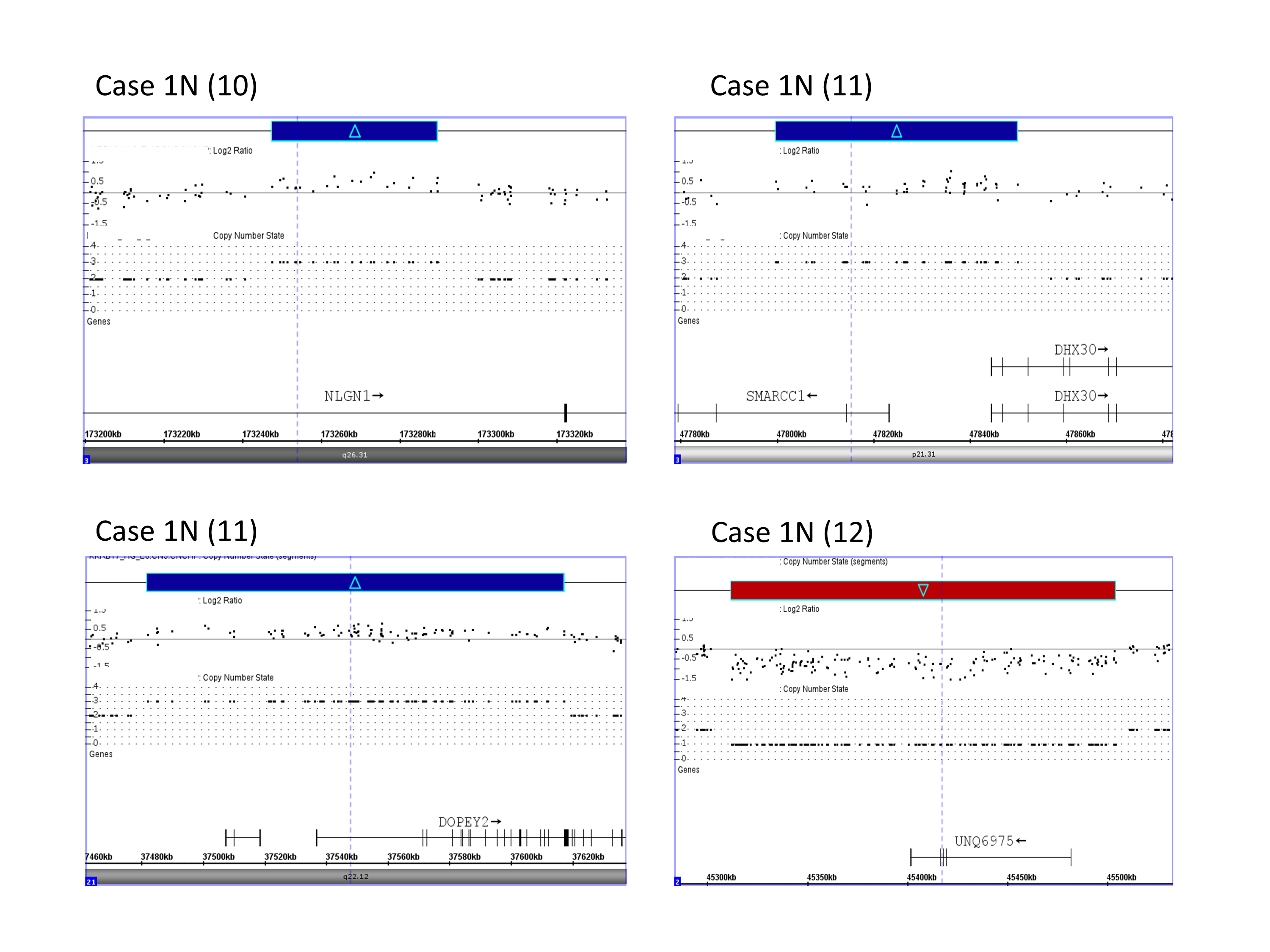

Supplement: Supplementary file 27 — (PNG 527 kb) [file 109_2020_1937_Fig19_ESM.png]

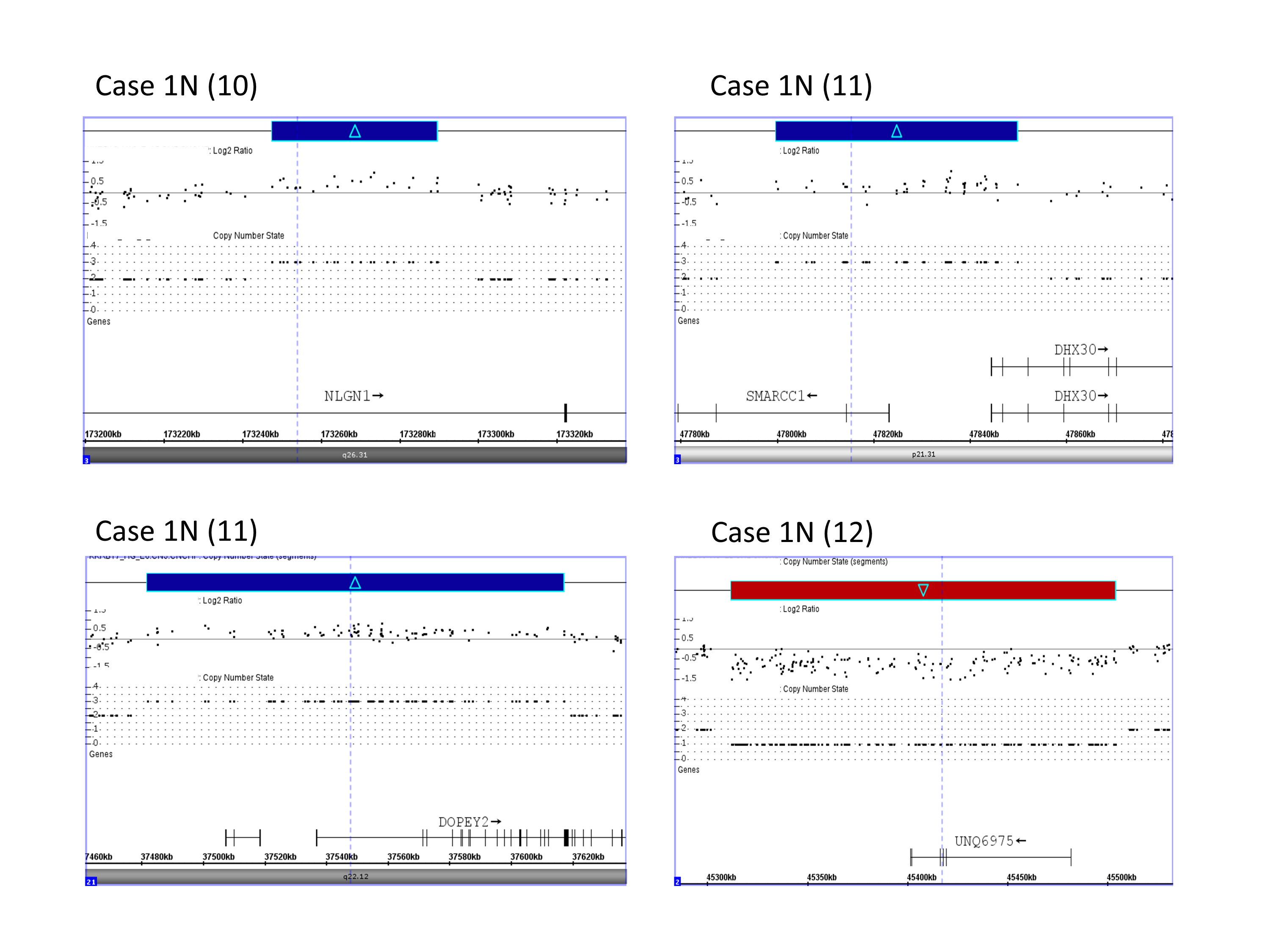

Supplement: Supplementary file 28 — High Resolution Image (TIF 620 kb) [file 109_2020_1937_MOESM15_ESM.tif]

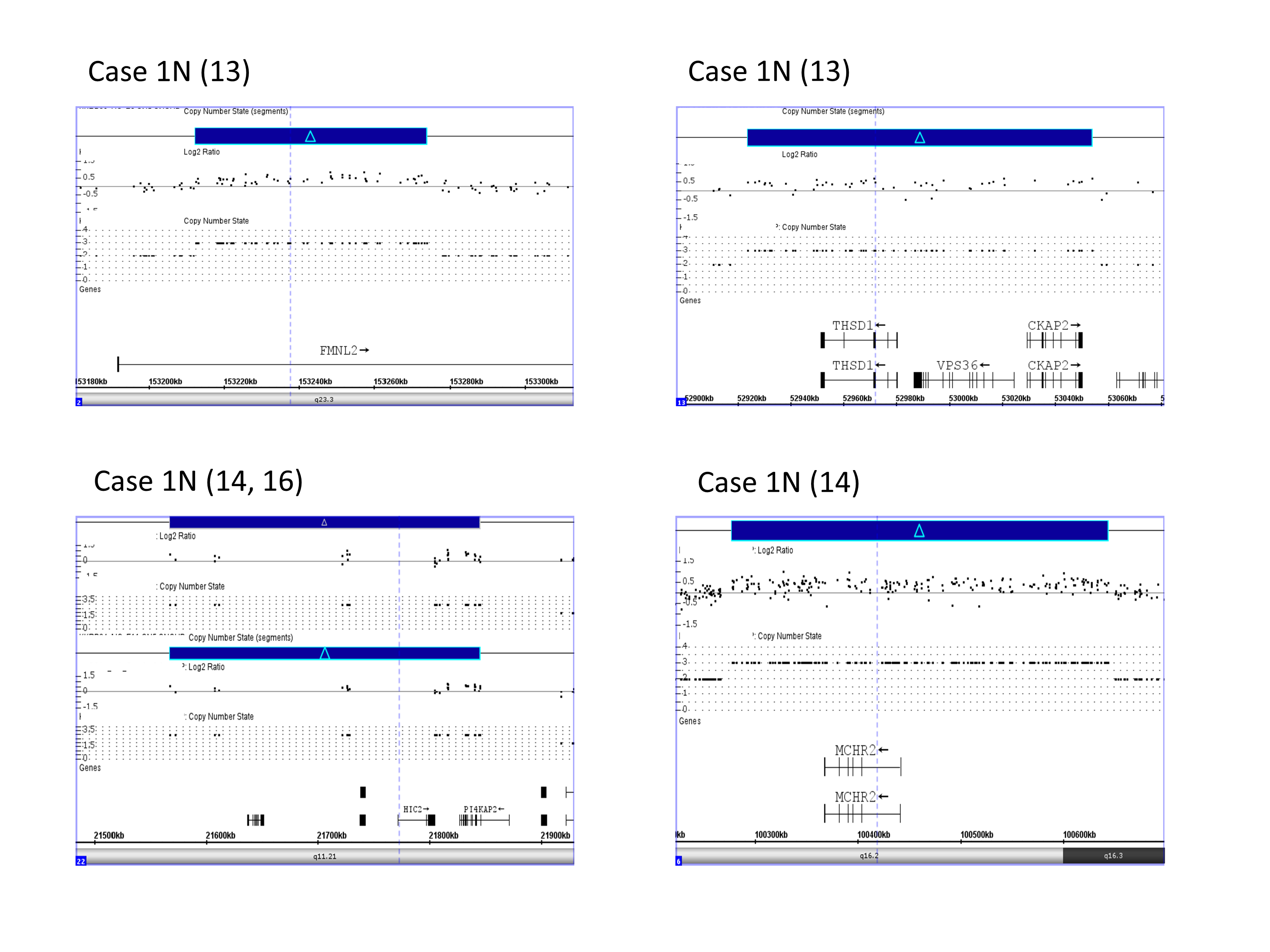

Supplement: Supplementary file 29 — (PNG 580 kb) [file 109_2020_1937_Fig20_ESM.png]

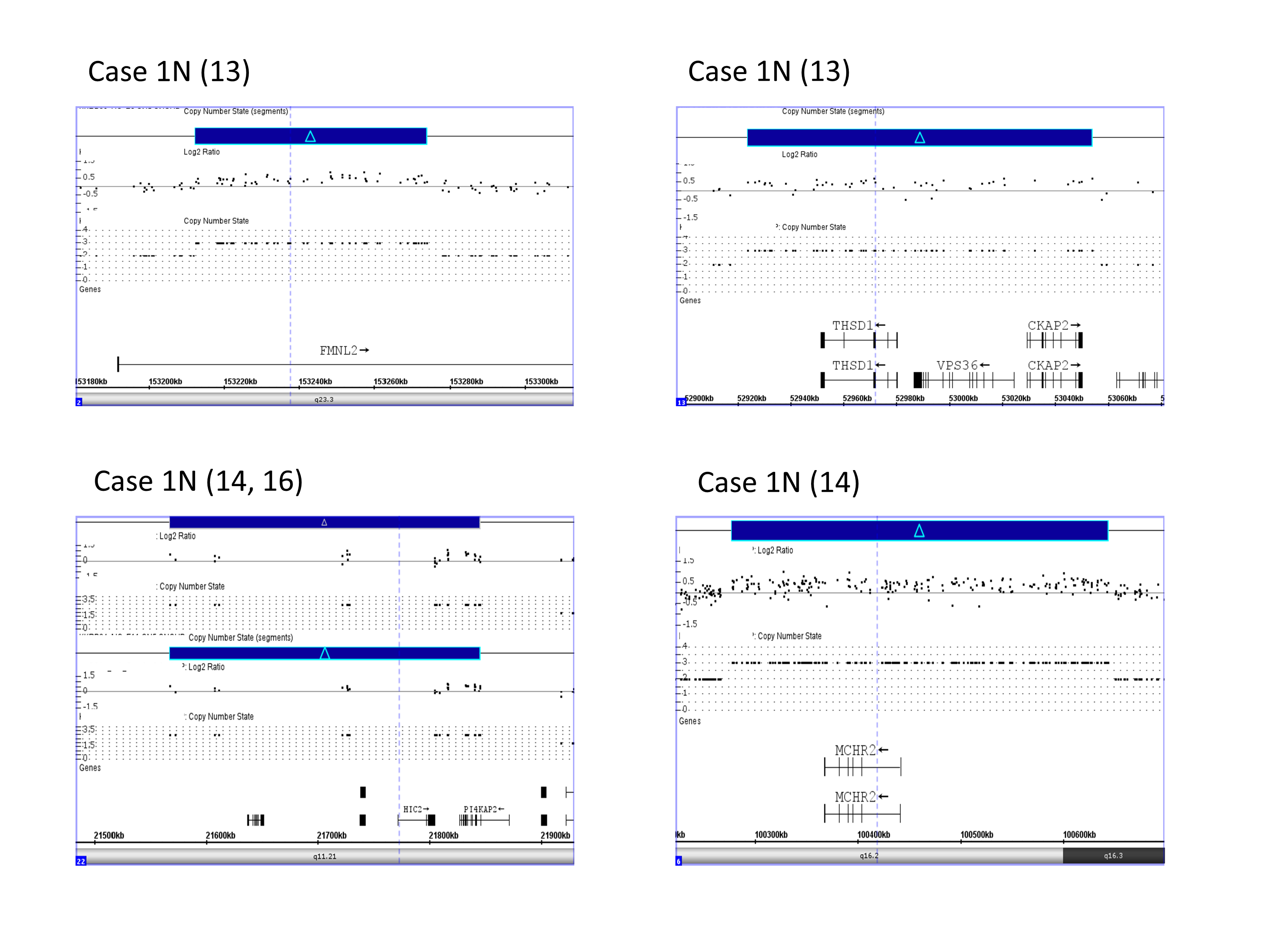

Supplement: Supplementary file 30 — High Resolution Image (TIF 659 kb) [file 109_2020_1937_MOESM16_ESM.tif]

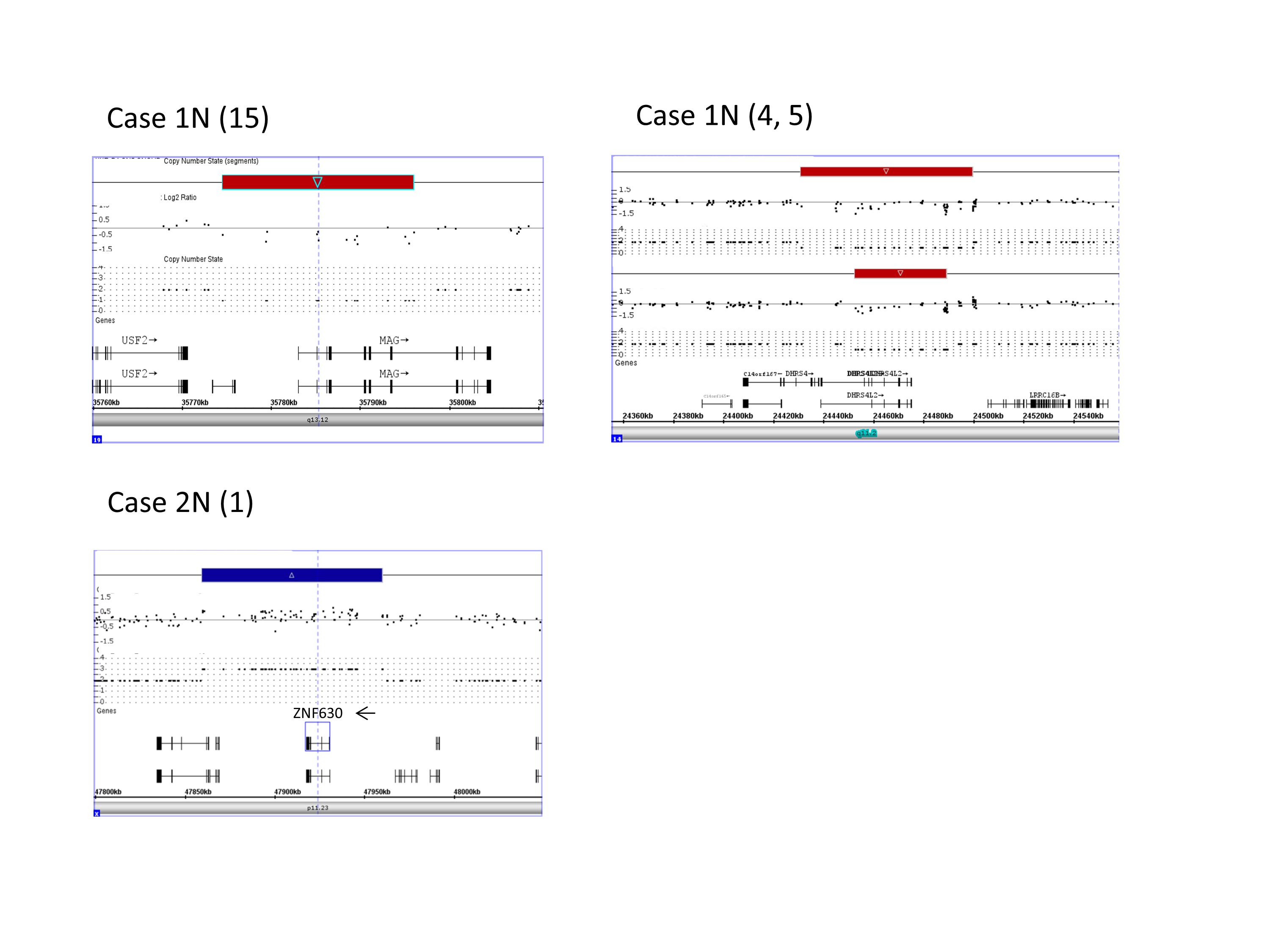

Supplement: Supplementary file 31 — (PNG 478 kb) [file 109_2020_1937_Fig21_ESM.png]

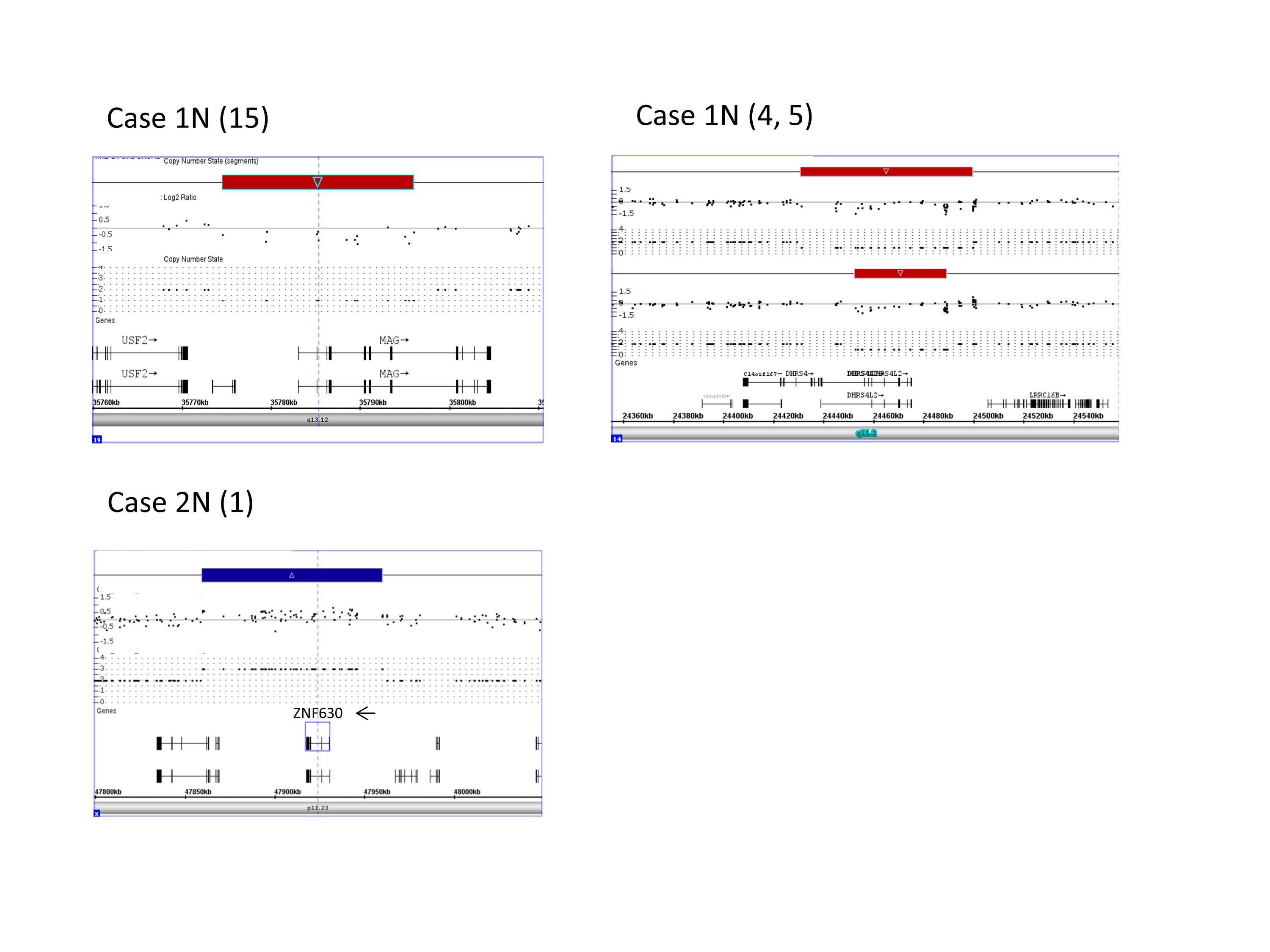

Supplement: Supplementary file 32 — High Resolution Image (TIF 515 kb) [file 109_2020_1937_MOESM17_ESM.tif]
