# Supplementary material for: Molecular karyotyping and gene expression analysis in childhood cancer patients
Source: J Mol Med (Berl). 2020 Jun 23;98(8):1107–23. doi: 10.1007/s00109-020-01937-4 (PMC7769790; doi:10.1007/s00109-020-01937-4)
Supplement: Supplementary file 33 — (DOCX 132 kb) [file 109_2020_1937_MOESM18_ESM.docx]

***Supplemental References***

***Introduction***

(Saam et al. 2015; Ding et al. 2005; Long et al. 2007; Khodarev et al. 2001; Amundson et al. 2008; Mirchia et al. 2019; Liang et al. 2016)

***Material and Methods***

(Bolger et al. 2014; Bastida-Lertxundi et al. 2014; Dobin et al. 2013; Liao et al. 2014; Robinson et al. 2010; R Core Team 2016; Law et al. 2014; Ritchie and Flicek 2015; Benjamini and Hochberg 1995; Ruijter et al. 2009; Ramon Goni 2009; Haaf 2000; Wirth et al. 1999)

***Results***

(Peng et al. 2017; Ko et al. 2008; Woo et al. 2017; Mazor et al. 2017; Peng et al. 2009; Tachiiri et al. 2006; Zhou et al. 2006; Zhou et al. 2007; Warters et al. 2009; Sprung et al. 2011; Song et al. 2018)

***Discussion***

(Krepischi et al. 2014; Krepischi et al. 2012; Kuiper et al. 2010; Kayser et al. 2018; Brown et al. 2012; Klimek and Tray 2016; Case et al. 2013; Bozic et al. 2010; Ciriello et al. 2013; Heinloth et al. 2003; Kis et al. 2006; Krille et al. 2012; Spycher et al. 2015; Boice, JR 1996; Budczies et al. 2017; Yang et al. 2017**)**

References

Amundson, Sally A.; Do, Khanh T.; Vinikoor, Lisa C.; Lee, R. Anthony; Koch-Paiz, Christine A.; Ahn, Jaeyong et al. (2008): Integrating global gene expression and radiation survival parameters across the 60 cell lines of the National Cancer Institute Anticancer Drug Screen. In *Cancer research* 68 (2), pp. 415–424. DOI: 10.1158/0008-5472.CAN-07-2120.

Bastida-Lertxundi, Nerea; López-López, Elixabet; Piñán, M. Angeles; Puiggros, Anna; Navajas, Aurora; Solé, Francesc; García-Orad, Africa (2014): Errors in the interpretation of copy number variations due to the use of public databases as a reference. In *Cancer genetics* 207 (4), pp. 164–167. DOI: 10.1016/j.cancergen.2014.03.001.

Benjamini, Yoav; Hochberg, Yosef (1995): Controlling the False Discovery Rate: A Practical and Powerful Approach to Multiple Testing. In *Journal of the Royal Statistical Society: Series B (Methodological)* 57 (1), pp. 289–300. DOI: 10.1111/j.2517-6161.1995.tb02031.x.

Boice, J. D., JR (1996): Cancer following irradiation in childhood and adolescence. In *Medical and pediatric oncology. Supplement* 1, pp. 29–34.

Bolger, Anthony M.; Lohse, Marc; Usadel, Bjoern (2014): Trimmomatic: a flexible trimmer for Illumina sequence data. In *Bioinformatics (Oxford, England)* 30 (15), pp. 2114–2120. DOI: 10.1093/bioinformatics/btu170.

Bozic, Ivana; Antal, Tibor; Ohtsuki, Hisashi; Carter, Hannah; Kim, Dewey; Chen, Sining et al. (2010): Accumulation of driver and passenger mutations during tumor progression. In *Proceedings of the National Academy of Sciences of the United States of America* 107 (43), pp. 18545–18550. DOI: 10.1073/pnas.1010978107.

Brown, J. R.; Hanna, M.; Tesar, B.; Pochet, N.; Vartanov, A.; Fernandes, S. M. et al. (2012): Germline copy number variation associated with Mendelian inheritance of CLL in two families. In *Leukemia* 26 (7), pp. 1710–1713. DOI: 10.1038/leu.2012.33.

Budczies, Jan; Denkert, Carsten; Győrffy, Balázs; Schirmacher, Peter; Stenzinger, Albrecht (2017): Chromosome 9p copy number gains involving PD-L1 are associated with a specific proliferation and immune-modulating gene expression program active across major cancer types. In *BMC medical genomics* 10 (1), p. 74. DOI: 10.1186/s12920-017-0308-8.

Case, Chanelle M.; Sackett, Dan L.; Wangsa, Danny; Karpova, Tatiana; McNally, James G.; Ried, Thomas; Camps, Jordi (2013): CKAP2 ensures chromosomal stability by maintaining the integrity of microtubule nucleation sites. In *PloS one* 8 (5), e64575. DOI: 10.1371/journal.pone.0064575.

Ciriello, Giovanni; Miller, Martin L.; Aksoy, Bülent Arman; Senbabaoglu, Yasin; Schultz, Nikolaus; Sander, Chris (2013): Emerging landscape of oncogenic signatures across human cancers. In *Nature genetics* 45 (10), pp. 1127–1133. DOI: 10.1038/ng.2762.

Ding, Liang-Hao; Shingyoji, Masato; Chen, Fanqing; Hwang, Jeng-Jong; Burma, Sandeep; Lee, Clarissa et al. (2005): Gene Expression Profiles of Normal Human Fibroblasts after Exposure to Ionizing Radiation: A Comparative Study of Low and High Doses. In *Radiation Research* 164 (1), pp. 17–26. DOI: 10.1667/RR3354.

Dobin, Alexander; Davis, Carrie A.; Schlesinger, Felix; Drenkow, Jorg; Zaleski, Chris; Jha, Sonali et al. (2013): STAR: ultrafast universal RNA-seq aligner. In *Bioinformatics (Oxford, England)* 29 (1), pp. 15–21. DOI: 10.1093/bioinformatics/bts635.

Haaf, Thomas (2000): Fluorescence In Situ Hybridization. In Robert A. Meyers (Ed.): Encyclopedia of analytical chemistry. Applications, theory and instrumentation, vol. 223. New York: Wiley, p. 582.

Heinloth, Alexandra N.; Shackelford, Rodney E.; Innes, Cynthia L.; Bennett, Lee; Li, Leping; Amin, Rupesh P. et al. (2003): Identification of distinct and common gene expression changes after oxidative stress and gamma and ultraviolet radiation. In *Molecular carcinogenesis* 37 (2), pp. 65–82. DOI: 10.1002/mc.10122.

Kayser, Katrin; Degenhardt, Franziska; Holzapfel, Stefanie; Horpaopan, Sukanya; Peters, Sophia; Spier, Isabel et al. (2018): Copy number variation analysis and targeted NGS in 77 families with suspected Lynch syndrome reveals novel potential causative genes. In *International journal of cancer* 143 (11), pp. 2800–2813. DOI: 10.1002/ijc.31725.

Khodarev, N. N.; Park, J. O.; Yu, J.; Gupta, N.; Nodzenski, E.; Roizman, B.; Weichselbaum, R. R. (2001): Dose-dependent and independent temporal patterns of gene responses to ionizing radiation in normal and tumor cells and tumor xenografts. In *Proceedings of the National Academy of Sciences of the United States of America* 98 (22), pp. 12665–12670. DOI: 10.1073/pnas.211443698.

Kis, Eniko; Szatmari, Tunde; Keszei, Marton; Farkas, Robert; Esik, Olga; Lumniczky, Katalin et al. (2006): Microarray analysis of radiation response genes in primary human fibroblasts. In *International journal of radiation oncology, biology, physics* 66 (5), pp. 1506–1514. DOI: 10.1016/j.ijrobp.2006.08.004.

Klimek, Virginia M.; Tray, Nancy J. (2016): Therapy-related myeloid neoplasms: what's in a name? In *Current opinion in hematology* 23 (2), pp. 161–166. DOI: 10.1097/MOH.0000000000000222.

Ko, Josephine M. Y.; Chan, Pui Ling; Yau, Wing Lung; Chan, Ho Kin; Chan, King Chi; Yu, Zhuo You et al. (2008): Monochromosome transfer and microarray analysis identify a critical tumor-suppressive region mapping to chromosome 13q14 and THSD1 in esophageal carcinoma. In *Molecular cancer research : MCR* 6 (4), pp. 592–603. DOI: 10.1158/1541-7786.MCR-07-0154.

Krepischi, Ana Cristina Victorino; Capelli, Leonardo Pires; Silva, Amanda Gonçalves; Araújo, Érica Sara Souza de; Pearson, Peter Lees; Heck, Benjamin et al. (2014): Large germline copy number variations as predisposing factor in childhood neoplasms. In *Future oncology (London, England)* 10 (9), pp. 1627–1633. DOI: 10.2217/fon.14.41.

Krepischi, Ana Cristina Victorino; Pearson, Peter Lees; Rosenberg, Carla (2012): Germline copy number variations and cancer predisposition. In *Future oncology (London, England)* 8 (4), pp. 441–450. DOI: 10.2217/fon.12.34.

Krille, Lucian; Zeeb, Hajo; Jahnen, Andreas; Mildenberger, Peter; Seidenbusch, Michael; Schneider, Karl et al. (2012): Computed tomographies and cancer risk in children: a literature overview of CT practices, risk estimations and an epidemiologic cohort study proposal. In *Radiation and environmental biophysics* 51 (2), pp. 103–111. DOI: 10.1007/s00411-012-0405-1.

Kuiper, Roland P.; Ligtenberg, Marjolijn J. L.; Hoogerbrugge, Nicoline; van Geurts Kessel, Ad (2010): Germline copy number variation and cancer risk. In *Current opinion in genetics & development* 20 (3), pp. 282–289. DOI: 10.1016/j.gde.2010.03.005.

Law, Charity W.; Chen, Yunshun; Shi, Wei; Smyth, Gordon K. (2014): voom: Precision weights unlock linear model analysis tools for RNA-seq read counts. In *Genome biology* 15 (2), R29. DOI: 10.1186/gb-2014-15-2-r29.

Liang, L.; Fang, J-Y; Xu, J. (2016): Gastric cancer and gene copy number variation: emerging cancer drivers for targeted therapy. In *Oncogene* 35 (12), pp. 1475–1482. DOI: 10.1038/onc.2015.209.

Liao, Yang; Smyth, Gordon K.; Shi, Wei (2014): featureCounts: an efficient general purpose program for assigning sequence reads to genomic features. In *Bioinformatics (Oxford, England)* 30 (7), pp. 923–930. DOI: 10.1093/bioinformatics/btt656.

Long, Xian-Hui; Zhao, Zeng-Qiang; He, Xing-Peng; Wang, Hui-Ping; Xu, Qin-Zhi; An, Jing et al. (2007): Dose-dependent expression changes of early response genes to ionizing radiation in human lymphoblastoid cells. In *International journal of molecular medicine* 19 (4), pp. 607–615.

Mazor, Tali; Chesnelong, Charles; Pankov, Aleksandr; Jalbert, Llewellyn E.; Hong, Chibo; Hayes, Josie et al. (2017): Clonal expansion and epigenetic reprogramming following deletion or amplification of mutant IDH1. In *Proceedings of the National Academy of Sciences of the United States of America* 114 (40), pp. 10743–10748. DOI: 10.1073/pnas.1708914114.

Mirchia, Kanish; Sathe, Adwait Amod; Walker, Jamie M.; Fudym, Yelena; Galbraith, Kristyn; Viapiano, Mariano S. et al. (2019): Total copy number variation as a prognostic factor in adult astrocytoma subtypes. In *Acta neuropathologica communications* 7 (1), p. 8. DOI: 10.1186/s40478-019-0746-y.

Peng, D. F.; Razvi, M.; Chen, H.; Washington, K.; Roessner, A.; Schneider-Stock, R.; El-Rifai, W. (2009): DNA hypermethylation regulates the expression of members of the Mu-class glutathione S-transferases and glutathione peroxidases in Barrett's adenocarcinoma. In *Gut* 58 (1), pp. 5–15. DOI: 10.1136/gut.2007.146290.

Peng, DunFa; Guo, Yan; Chen, Heidi; Zhao, Shilin; Washington, Kay; Hu, TianLing et al. (2017): Integrated molecular analysis reveals complex interactions between genomic and epigenomic alterations in esophageal adenocarcinomas. In *Scientific reports* 7, p. 40729. DOI: 10.1038/srep40729.

R Core Team (2016): R: A language and environment for statistical computing. R Foundation for Statistical Computing, Vienna, Austria. URL http://www.R-project.org/.

Ramon Goni (2009): Microsoft Word - Integromics qPCR Statistics. In *Intergromic White Paper*. Available online at https://gene-quantification.de/integromics-qpcr-statistics-white-paper.pdf, checked on 7/24/2019.

Ritchie, Graham R. S.; Flicek, Paul (2015): Functional Annotation of Rare Genetic Variants. In Eleftheria Zeggini, Andrew Morris (Eds.): Assessing Rare Variation in Complex Traits. Design and Analysis of Genetic Studies. 1st ed. 2015. New York, NY: Springer, pp. 57–70.

Robinson, Mark D.; McCarthy, Davis J.; Smyth, Gordon K. (2010): edgeR: a Bioconductor package for differential expression analysis of digital gene expression data. In *Bioinformatics (Oxford, England)* 26 (1), pp. 139–140. DOI: 10.1093/bioinformatics/btp616.

Ruijter, J. M.; Ramakers, C.; Hoogaars, W. M. H.; Karlen, Y.; Bakker, O.; van den Hoff, M. J. B.; Moorman, A. F. M. (2009): Amplification efficiency: linking baseline and bias in the analysis of quantitative PCR data. In *Nucleic acids research* 37 (6), e45. DOI: 10.1093/nar/gkp045.

Saam, Jennifer; Moyes, Kelsey; Landon, Michelle; Williams, Kayon; Kaldate, Rajesh R.; Arnell, Christopher; Wenstrup, Richard (2015): Hereditary cancer-associated mutations in women diagnosed with two primary cancers: an opportunity to identify hereditary cancer syndromes after the first cancer diagnosis. In *Oncology* 88 (4), pp. 226–233. DOI: 10.1159/000368836.

Song, Jianyuan; Zhang, Huojun; Wang, Zhenyu; Xu, Wanglei; Zhong, Li; Cao, Jinming et al. (2018): The Role of FABP5 in Radiation-Induced Human Skin Fibrosis. In *Radiation Research* 189 (2), pp. 177–186. DOI: 10.1667/RR14901.1.

Sprung, Carl N.; Li, Jason; Hovan, Daniel; McKay, Michael J.; Forrester, Helen B. (2011): Alternative transcript initiation and splicing as a response to DNA damage. In *PloS one* 6 (10), e25758. DOI: 10.1371/journal.pone.0025758.

Spycher, Ben D.; Lupatsch, Judith E.; Zwahlen, Marcel; Röösli, Martin; Niggli, Felix; Grotzer, Michael A. et al. (2015): Background ionizing radiation and the risk of childhood cancer: a census-based nationwide cohort study. In *Environmental health perspectives* 123 (6), pp. 622–628. DOI: 10.1289/ehp.1408548.

Tachiiri, Seiji; Katagiri, Toyomasa; Tsunoda, Tatsuhiko; Oya, Natsuo; Hiraoka, Masahiro; Nakamura, Yusuke (2006): Analysis of gene-expression profiles after gamma irradiation of normal human fibroblasts. In *International journal of radiation oncology, biology, physics* 64 (1), pp. 272–279. DOI: 10.1016/j.ijrobp.2005.08.030.

Warters, Raymond L.; Packard, Ann T.; Kramer, Gwen F.; Gaffney, David K.; Moos, Philip J. (2009): Differential gene expression in primary human skin keratinocytes and fibroblasts in response to ionizing radiation. In *Radiation Research* 172 (1), pp. 82–95. DOI: 10.1667/RR1677.1.

Wirth, J.; Nothwang, H. G.; van der Maarel, S.; Menzel, C.; Borck, G.; Lopez-Pajares, I. et al. (1999): Systematic characterisation of disease associated balanced chromosome rearrangements by FISH: cytogenetically and genetically anchored YACs identify microdeletions and candidate regions for mental retardation genes. In *Journal of medical genetics* 36 (4), pp. 271–278.

Woo, Hyun Goo; Choi, Ji-Hye; Yoon, Sarah; Jee, Byul A.; Cho, Eun Ju; Lee, Jeong-Hoon et al. (2017): Integrative analysis of genomic and epigenomic regulation of the transcriptome in liver cancer. In *Nature communications* 8 (1), p. 839. DOI: 10.1038/s41467-017-00991-w.

Yang, Lu; Wang, Ya-Zhe; Zhu, Hong-Hu; Chang, Yan; Li, Ling-Di; Chen, Wen-Min et al. (2017): PRAME Gene Copy Number Variation Is Related to Its Expression in Multiple Myeloma. In *DNA and cell biology* 36 (12), pp. 1099–1107. DOI: 10.1089/dna.2017.3951.

Zhou, Tong; Chou, Jeff; Mullen, Thomas E.; Elkon, Rani; Zhou, Yingchun; Simpson, Dennis A. et al. (2007): Identification of primary transcriptional regulation of cell cycle-regulated genes upon DNA damage. In *Cell cycle (Georgetown, Tex.)* 6 (8), pp. 972–981. DOI: 10.4161/cc.6.8.4106.

Zhou, Tong; Chou, Jeff W.; Simpson, Dennis A.; Zhou, Yingchun; Mullen, Thomas E.; Medeiros, Margarida et al. (2006): Profiles of global gene expression in ionizing-radiation-damaged human diploid fibroblasts reveal synchronization behind the G1 checkpoint in a G0-like state of quiescence. In *Environmental health perspectives* 114 (4), pp. 553–559. DOI: 10.1289/ehp.8026.
